# Quark flavour observables in the Littlest Higgs model with T-parity after LHC Run 1

**DOI:** 10.1140/epjc/s10052-016-4019-7

**Published:** 2016-04-02

**Authors:** Monika Blanke, Andrzej J. Buras, Stefan Recksiegel

**Affiliations:** 1 CERN Theory Division, 1211 Geneva 23, Switzerland; 2 Institut für Theoretische Teilchenphysik, Karlsruhe Institute of Technology, Engesserstraße 7, 76128 Karlsruhe, Germany; 3 Institut für Kernphysik, Karlsruhe Institute of Technology, Hermann-von-Helmholtz-Platz 1, 76344 Eggenstein-Leopoldshafen, Germany; 4 TUM Institute for Advanced Study, Lichtenbergstr. 2a, 85747 Garching, Germany; 5 Physik Department, Technische Universität München, James-Franck-Straße, 85747 Garching, Germany

## Abstract

The Littlest Higgs model with T-parity (LHT) belongs to the simplest new physics scenarios with new sources of flavour and CP violation. The latter originate in the interactions of ordinary quarks and leptons with heavy mirror quarks and leptons that are mediated by new heavy gauge bosons. Also a heavy fermionic top partner is present in this model which communicates with the SM fermions by means of standard $W^\pm$ and $Z^0$ gauge bosons. We present a new analysis of quark flavour observables in the LHT model in view of the oncoming flavour precision era. We use all available information on the CKM parameters, lattice QCD input and experimental data on quark flavour observables and corresponding theoretical calculations, taking into account new lower bounds on the symmetry breaking scale and the mirror quark masses from the LHC. We investigate by how much the branching ratios for a number of rare *K* and *B* decays are still allowed to depart from their SM values. This includes $K^+\rightarrow \pi ^+\nu \bar{\nu }$, $K_{L}\rightarrow \pi ^0\nu \bar{\nu }$, $K_L\rightarrow \mu ^+\mu ^-$, $B\rightarrow X_s\gamma$, $B_{s,d}\rightarrow \mu ^+\mu ^-$, $B\rightarrow K^{(*)}\ell ^+\ell ^-$, $B\rightarrow K^{(*)}\nu \bar{\nu }$, and $\varepsilon '/\varepsilon$. Taking into account the constraints from $\Delta F=2$ processes, significant departures from the SM predictions for $K^+\rightarrow \pi ^+\nu \bar{\nu }$ and $K_{L}\rightarrow \pi ^0\nu \bar{\nu }$ are possible, while the effects in *B* decays are much smaller. In particular, the LHT model favours $\mathcal {B}(B_{s}\rightarrow \mu ^+\mu ^-) \ge \mathcal {B}(B_{s}\rightarrow \mu ^+\mu ^-)_\mathrm{SM}$, which is not supported by the data, and the present anomalies in $B\rightarrow K^{(*)}\ell ^+\ell ^-$ decays cannot be explained in this model. With the recent lattice and large *N* input the imposition of the $\varepsilon '/\varepsilon$ constraint implies a significant suppression of the branching ratio for $K_{L}\rightarrow \pi ^0\nu \bar{\nu }$ with respect to its SM value while allowing only for small modifications of $K^+\rightarrow \pi ^+\nu \bar{\nu }$. Finally, we investigate how the LHT physics could be distinguished from other models by means of indirect measurements and discuss the consequences for quark flavour observables of not finding any LHT state in the coming years.

## Introduction

Elementary Particle Physics stands at the threshold of big discoveries. The completion of the Standard Model (SM) through the Higgs discovery in 2012 [1, 2] has shown that we are on the right track towards the fundamental theory. But there is a common belief that in order to understand the nature around us new particles and new forces are required. Fortunately in the coming years the ATLAS and CMS experiments will tell us directly whether new physics (NP) is present up to scales as high as several $\, \mathrm{TeV}$. These efforts will be accompanied by the indirect search for NP with the help of quantum fluctuations. This indirect route to short-distance scales will be followed in this decade by several experiments [3], in particular the LHCb experiment and to some extent by CMS and ATLAS through more precise data on rare $B_{s,d}$ decays and CP violation. But equally important are the dedicated kaon experiments NA62 at CERN and KOPIO at J-PARC and the Belle II experiment at SuperKEKB. Also the study of charged lepton flavour violation and of electric dipole moments at various laboratories will be very important in this respect.

One of the important questions in this context is whether the framework of constrained Minimal Flavour Violation (CMFV) [4–6] and the more general framework of MFV [7] will be capable of describing the future data. In models of this class, when flavour blind phases are absent or set to zero, stringent relations between various observables in the *K*, $B_d^0$ and $B_s^0$ systems are present [5]. Consequently the departures from SM expectations in this class of models in these three meson systems are correlated with each other, allowing very transparent tests of these simple NP scenarios. However, generally these relations can be strongly violated, implying often other correlations between observables characteristic for a given NP scenario. Such correlations, being less sensitive to the model parameters than individual observables, can often allow a transparent distinction between various models proposed in the literature [8].

Among the simplest extensions of the SM that go beyond the concept of MFV is the Littlest Higgs Model with T-parity (LHT) [9–13]. In this model, new heavy fermions and gauge bosons are present. The interactions of ordinary quarks and leptons with these new heavy mirror quarks and leptons, mediated by new heavy electroweak gauge bosons, introduce new sources of flavour and CP violation. The most characteristic signals of these new interactions are violations of CMFV and MFV relations between observables in different meson systems. At the same time, no new effective operators are generated beyond those which are already present in the SM. Therefore non-perturbative uncertainties are not increased with respect to the ones present in the SM. This operator structure can be tested by studying correlations between observables from the same meson system.

In the last decade we have performed a number of extensive phenomenological analyses of the LHT model [14–21]. Further phenomenological discussions of flavour in the LHT model can be found in [22–24]. Our 2009 analysis in [21] has shown that significant deviations from SM expectations were possible in the LHT model at that time. Our main findings in 2009, related to quark flavour physics, can be summarized as follows:

- The CMFV relations between *K*, $B_d$ and $B_s$ systems can be strongly violated. This allowed one to remove the tension between $\varepsilon _K$ and $S_{\psi K_S}$ [25–29].
- Interestingly, in the LHT model it was not possible to obtain the mixing induced CP-asymmetry $S_{\psi \phi }$ of $\mathcal {O}(1)$ and values above 0.3 were very unlikely. In fact the most recent data from LHCb [30] confirm this prediction. Yet the LHT model can both enhance or suppress $S_{\psi \phi }$ w. r. t. its SM value. As we will stress below this could provide an important distinction from other models, like the Two Higgs Doublet model with MFV and flavour blind phases ($\mathrm{2HDM_{\overline{MFV}}}$) [31, 32] where $S_{\psi \phi }$ can only be enhanced due to its correlation with $S_{\psi K_S}$.
- $\mathcal {B}(K_{L}\rightarrow \pi ^0\nu \bar{\nu })$ and $\mathcal {B}(K^+\rightarrow \pi ^+\nu \bar{\nu })$ could be enhanced by factors of 3 and 2.5, respectively, but not simultaneously with $S_{\psi \phi }$. Also, a distinctive correlation between these two branching ratios, typical for models with only SM operators [33], holds.
- Rare $B_{s,d}$ decays turned out to be SM-like but still some measurable departures from SM predictions were possible. In particular $\mathcal {B}(B_{s,d}\rightarrow \mu ^+\mu ^-)$ could be enhanced by $30\,\%$, with a significant part of this enhancement coming from the T-even sector.

In view of the oncoming flavour precision era it is of interest to update our 2009 analysis, as during the last 6 years substantial improvements on both experimental and theoretical inputs have been achieved. In particular:

- The data from ATLAS and CMS, both on Higgs physics and on direct NP searches, provide important constraints on the LHT parameter space. Further significant improvements can be expected from LHC Run 2. In particular in our 2009 analysis we had restricted the mirror quark masses to lie below the $1\, \mathrm{TeV}$ scale, in order to make them easily accessible to direct searches. The absence of a signal in run 1 of the LHC, however, pushes the masses of these fermions to heavier ranges [34]. As we will see below this change has a significant impact on the possible size of LHT effects in rare decays.
- The values of CKM parameters extracted from tree-level decays are presently better constrained and will be significantly improved in the coming years.
- Significant progress has been made by the lattice community in calculating various parameters like weak decay constants and non-perturbative $B_i$ parameters.
- The mixing induced CP-asymmetry $S_{\psi \phi }$ is presently known with much higher accuracy than in 2009.
- The branching ratio $\mathcal {B}(B_{s}\rightarrow \mu ^+\mu ^-)$ has been found SM-like, as expected within the LHT model, but significant NP contributions are still allowed due to the large experimental uncertainty and to a lesser extent parametric uncertainties dominantly present in the value of $|V_{cb}|$. Still, the improved precision on the SM prediction for $\mathcal {B}(B_{s}\rightarrow \mu ^+\mu ^-)$ makes a detailed comparison of theory and data possible.
- The data on $B\rightarrow K^{(*)}\ell ^+\ell ^-$ from LHCb provided a new arena for testing the LHT model. In fact, it will turn out that the LHT model is unable to describe this new data.
- The measured values of the ratios *R*(*D*) and $R(D^*)$ show a $3.9\sigma$ deviation from their SM predictions [35]. We will investigate whether the LHT model could be the origin of this discrepancy. Note that these ratios have not been considered in the context of the LHT model before.
- The new results for the non-perturbative parameters $B_6^{(1/2)}$ and $B_8^{(3/2)}$ from lattice QCD [36, 37] and the large *N* approach [38] imply that $\varepsilon '/\varepsilon$ in the SM is significantly below the data [39]. The question arises whether the LHT model could help in solving this problem.
- Very importantly the NA62 experiment at CERN should provide in the next years a new measurement of $\mathcal {B}(K^+\rightarrow \pi ^+\nu \bar{\nu })$, which will be an important test of the LHT model in view of very small theoretical uncertainties in this decay.

In view of these developments the two main goals of our present analysis are:

- We confront the rich pattern of flavour violation in this model with the present data and investigate the allowed size of new flavour-violating effects, taking present bounds and improved input into account.
- We investigate what size of new flavour-violating effects will still be possible if we do not find any LHT state during the next LHC run. This means setting the masses of new gauge bosons and mirror quarks to be several TeV.

Our paper is organized as follows. In Sect. 2 we recall basic features of the LHT model that are relevant to understand our analysis. In particular, we recall the flavour structure of this model. Due to the absence of new operators, the full quark flavour analysis can be formulated in terms of a number of one-loop master functions. We refrain from repeating the complete formulae for these functions in the LHT model that can all be found in our previous papers. But in Sect. 3 we collect the relevant expressions for quark flavour observables that can be compactly written in terms of these master functions. This will allow us to indicate the changes in the CKM input and in non-perturbative parameters as well as QCD corrections that took place since our 2009 analysis. Section 4 is devoted to a brief review of the direct constraints on the LHT parameter space, implied by the available data from ATLAS and CMS. In Sect. 5, after presenting our strategy for the numerical analysis and summarizing the input, we present the results for a multitude of observables in the quark sector. The highlights of our analysis are listed in Sect. 6, where we also present a brief outlook for the coming years.

## General structure of the LHT model

### Preliminaries

The Littlest Higgs model without [11] T-parity has been invented to solve the problem of the quadratic divergences in the Higgs mass without using supersymmetry. In this approach the cancellation of divergences in $$ m_H$$ is achieved with the help of new particles of the same spin-statistics. Basically the SM Higgs is kept light because it is a pseudo-Goldstone boson of a spontaneously broken global symmetry:

1 $$\begin{aligned} SU(5)\rightarrow SO(5). \end{aligned}$$

Thus the Higgs mass is protected by a global symmetry. In order to achieve this the gauge group has to be extended to

2 $$\begin{aligned} G_\mathrm{LHT}=SU(3)_c\times [SU(2)\times U(1)]_1\times [SU(2)\times U(1)]_2 \end{aligned}$$

and the symmetry breaking mechanism has to be properly arranged (*collective symmetry breaking*). Excellent reviews of Little Higgs models can be found in [40, 41].

### Particle content of the LHT model

In order to make the Littlest Higgs model consistent with electroweak precision tests and simultaneously have the new particles of this model in the reach of the LHC, a discrete symmetry, T-parity, has been introduced [12, 13]. Under T-parity all SM particles are *even*. Among the new particles only a heavy $Q=+2/3$ charged top partner quark, called $T_+$, belongs to the even sector. Its role is to cancel the quadratic divergence in the Higgs mass generated by the ordinary top quark. The even sector and also the model without T-parity belong to the CMFV class if only flavour violation in the down-quark sector is considered [42, 43].

More interesting from the point of view of FCNC processes in the quark sector is the T-odd sector. It contains three doublets of mirror quarks,

3 $$\begin{aligned} \begin{pmatrix} u^1_{H} \\ d^1_{H} \end{pmatrix}\!,\quad \begin{pmatrix} u^2_{H} \\ d^2_{H} \end{pmatrix}\!,\quad \begin{pmatrix} u^3_{H} \\ d^3_{H} \end{pmatrix}\!. \end{aligned}$$

To first order in *v* / *f*, with $f=\mathcal {O}(1\, \mathrm{TeV})$, the mirror quarks have vectorial couplings under $SU(2)_L\times U(1)_Y$ and their masses satisfy

4 $$\begin{aligned} m^u_{H1}=m^d_{H1},\quad m^u_{H2}=m^d_{H2},\quad m^u_{H3}=m^d_{H3}. \end{aligned}$$

Mirror quarks communicate with the SM quarks by means of heavy gauge bosons

5 $$\begin{aligned} W_H^\pm ,\, Z_H,\, A_H, \end{aligned}$$

which can be considered as “partners” of the SM gauge bosons. They are T-odd particles with masses given to lowest order in *v* / *f* by

6 $$\begin{aligned} M_{W_H}=M_{Z_H}=gf,\quad M_{A_H}=\frac{g'f}{\sqrt{5}}=\frac{\tan {\theta _W}}{\sqrt{5}}M_{W_H}\simeq \frac{M_{W_H}}{4.1}, \end{aligned}$$

where *g* and $g'$ are the usual couplings of $SU(2)_L$ and $U(1)_Y$, respectively.

### Flavour structure of the LHT model

The interactions between ordinary down quarks and mirror quarks, mediated by gauge bosons $W_H^\pm \,$, $Z_H\,$, $A_H$, are governed by the new mixing matrix $V_{Hd}$. The corresponding matrix $V_{Hu}$ in the up sector is obtained by means of the relation [22, 44]

7 $$\begin{aligned} V_{Hu}^\dagger V_{Hd}^{\,}=V^{}_\text {CKM}. \end{aligned}$$

Thus we have new flavour- and CP-violating contributions to decay amplitudes in this model. These new interactions can have a structure that is very different from the CKM matrix.

The difference between the CMFV models and the LHT model can be transparently seen in the formulation of FCNC processes in terms of the master one-loop functions that multiply the CKM factors $\lambda _t^{(i)}$

8 $$\begin{aligned} \lambda _t^{(K)} = V_{ts}^*\,V_{td},\quad \lambda _t^{(d)} = V_{tb}^*\,V_{td},\quad \lambda _t^{(s)} = V_{tb}^*\,V_{ts}, \end{aligned}$$

for *K*, $B_d$ and $B_s$ systems, respectively. This formulation can be used straightforwardly here because the LHT model has the same operator structure as the SM and the models with CMFV, except that the real and universal master functions of the latter models become complex quantities and the property of the flavour universality of these functions is lost. Consequently the usual CMFV relations between *K*, $B_d$ and $B_s$ systems are generally broken.

Explicitly, the new functions in the LHT model are given as follows ($i=K,d,s$):

9 $$\begin{aligned} S_i= & {} S_\text {SM} + \bar{S}_\text {even} + \frac{1}{\lambda _t^{(i)}} \bar{S}_i^\text {odd} \quad \equiv \quad |S_i|\, e^{i\, \theta _S^i}, \end{aligned}$$

10 $$\begin{aligned} X_i= & {} X_\text {SM} + \bar{X}_\text {even} + \frac{1}{\lambda _t^{(i)}} \bar{X}_i^\text {odd} \quad \equiv \quad |X_i|\, e^{i\, \theta _X^i}, \end{aligned}$$

11 $$\begin{aligned} Y_i= & {} Y_\text {SM} + \bar{Y}_\text {even} + \frac{1}{\lambda _t^{(i)}} \bar{Y}_i^\text {odd} \quad \equiv \quad |Y_i|\, e^{i\, \theta _Y^i}, \end{aligned}$$

12 $$\begin{aligned} Z_i= & {} Z_\text {SM} + \bar{Z}_\text {even} + \frac{1}{\lambda _t^{(i)}} \bar{Z}_i^\text {odd} \quad \equiv \quad |Z_i|\, e^{i\, \theta _Z^i}. \end{aligned}$$

Here $S_\text {SM}$, $X_\text {SM}$, $Y_\text {SM}$ and $Z_\text {SM}$ are the SM contributions for which explicit expressions can be found in [8]. $\bar{S}_\text {even}$, $\bar{X}_\text {even}$, $\bar{Y}_\text {even}$ and $\bar{Z}_\text {even}$ are the contributions from the T-even sector, that is, the contributions of $T_+$ and of *t* at order $v^2/f^2$ necessary to make the GIM mechanism work. The latter contributions, similarly to $S_\text {SM}$, $X_\text {SM}$, $Y_\text {SM}$ and $Z_\text {SM}$, are real and independent of $i=K, d, s$. Explicit expressions for them can be found in [14].

The functions $\bar{S}_i^\text {odd}$, $\bar{X}_i^\text {odd}$, $\bar{Y}_i^\text {odd}$ and $\bar{Z}_i^\text {odd}$ represent the T-odd sector of the LHT model and are obtained from penguin and box diagrams with internal mirror quarks and new gauge bosons. Explicit expressions for these functions can be found in our previous papers [14, 15, 21] and will not be repeated here.

At this point it should be recalled that in our earlier papers, when calculating $\bar{X}_i^\text {odd}$, $\bar{Y}_i^\text {odd}$ and $\bar{Z}_i^\text {odd}$, we had overlooked an $\mathcal {O}(v^2/f^2)$ contribution to the $Z^0$-penguin diagrams. This contribution has been identified by Goto et al. [23] in the context of their study of the $K\rightarrow \pi \nu \bar{\nu }$ decays in the LHT model, and independently by del Aguila et al. [24] in the context of the corresponding analysis of the LFV decays $\mu \rightarrow e\gamma$ and $\mu \rightarrow 3e$. At the same time, these authors have confirmed our calculations except for the omission mentioned above. The corrected Feynman rules of [15] implied by the findings of [23, 24] are collected in Appendix A in [21]. In that paper also the implied shifts in the corresponding *Z*-penguin functions and consequently in $\bar{X}_i^\text {odd}$, $\bar{Y}_i^\text {odd}$ and $\bar{Z}_i^\text {odd}$ are given.

A review on flavour physics in the LHT model can be found in [45] and selected papers containing details of the pattern of flavour violation in this model can be found in [14–16, 21, 23, 24, 33].

### LHT as a representative example

Before moving on, we address the question whether our results remain valid in the more general context of Little Higgs models with T-parity, independent of the details of the Littlest Higgs model.^1^ The flavour-violating effects in the LHT model found by us are mostly due to the T-odd sector of the model, namely the heavy electroweak gauge bosons and mirror fermions, with only left-handed couplings to the SM quarks and leptons. The presence of these states is generic to the class of Little Higgs models with T-parity. Some details, like the precise form of the mirror quark coupling to the standard *Z* boson, are indeed model dependent, rendering a general quantitative analysis of the whole class of Little Higgs models with T-parity impossible. However, we point out that the overall structure of effects remains unaffected. We therefore expect our results to hold, at least qualitatively, beyond the concrete and rather restricted framework of the LHT model.

**Table 1:** Values of the experimental and theoretical quantities used as input parameters as of July 2015. For future updates see PDG [81], FLAG [79] and HFAG [35]

$G_F = 1.16638(1)\cdot 10^{-5}\, \mathrm{GeV}^{-2}$ [81]	$m_{B_d}=5279.58(17)\, \mathrm{MeV}$ [81]
$M_W = 80.385(15) \, \mathrm{GeV}$ [81]	$m_{B_s} = 5366.8(2)\, \mathrm{MeV}$ [81]
$\sin ^2\theta _W = 0.23126(13)$ [81]	$F_{B_d} = 190.5(42)\, \mathrm{MeV}$ [79]
$\alpha (M_Z) = 1/127.9$ [81]	$F_{B_s} = 227.7(45)\, \mathrm{MeV}$ [79]
$\alpha _s(M_Z)= 0.1185(6)$ [81]	$\hat{B}_{B_d} =1.27(10)$, ${\hat{B}}_{B_s} =1.33(6)$ [79]
$m_u(2\, \mathrm{GeV})=2.16(11)\, \mathrm{MeV}$ [79]	$\hat{B}_{B_s}/\hat{B}_{B_d} = 1.06(11)$ [79]
$m_d(2\, \mathrm{GeV})=4.68(16)\, \mathrm{MeV}$ [79]	$F_{B_d} \sqrt{\hat{B}_{B_d}} = 216(15)\, \mathrm{MeV}$ [79]
$m_s(2\, \mathrm{GeV})=93.8(24) \, \mathrm{MeV}$ [79]	$F_{B_s} \sqrt{\hat{B}_{B_s}} = 266(18)\, \mathrm{MeV}$ [79]
$m_c(m_c) = 1.275(25) \, \mathrm{GeV}$ [81]	$\xi = 1.268(63)$ [79]
$m_b(m_b)=4.18(3)\, \mathrm{GeV}$ [81]	$\eta _B=0.55(1)$ [96, 97]
$m_t(m_t) = 163(1)\, \mathrm{GeV}$ [98, 99]	$\Delta M_d = 0.510(3) \,\text {ps}^{-1}$ [35]
$m_K= 497.614(24)\, \mathrm{MeV}$ [81]	$\Delta M_s = 17.757(21) \,\text {ps}^{-1}$ [35]
$F_K = 156.1(11)\, \mathrm{MeV}$ [98]	$S_{\psi K_S}= 0.691(17)$ [35]
$\hat{B}_K= 0.750(15)$ [79, 80]	$S_{\psi \phi }= 0.015(35)$ [35]
$\kappa _\epsilon =0.94(2)$ [26, 46]	$\Delta \Gamma _s/\Gamma _s=0.122(9)$ [35]
$\eta _{cc}=1.87(76)$ [100]	$\tau _{B_s}= 1.509(4)\,\text {ps}$ [35]
$\eta _{tt}=0.5765(65)$ [96]	$\tau _{B_d}= 1.520(4) \,\text {ps}$ [35]
$\eta _{ct}= 0.496(47)$ [101]	$\tau _{B^\pm }= 1.638(4)\,\text {ps}$ [35]
$\Delta M_K= 0.5293(9)\cdot 10^{-2} \,\text {ps}^{-1}$ [81]	$|V_{us}|=0.2253(8)$ [81]
$|\epsilon _K|= 2.228(11)\cdot 10^{-3}$ [81]	$\gamma =(73.2^{+6.3}_{-7.0})^\circ$ [102]
$|V^{\mathrm{avg}}_{cb}|=40.7(14)\cdot 10^{-3}$ [69]	$|V^{\mathrm{avg}}_{ub}|=3.88(29)\cdot 10^{-3}$ [69]

## Basic formulae for quark flavour observables

### $\Delta F=2$ observables

The flavour parameters of the quark sector in the LHT model are first of all bounded by very precise data on

13 $$\begin{aligned} \Delta M_s, \quad \Delta M_d, \quad \varepsilon _K, \end{aligned}$$

but also by the data on the mixing induced CP-asymmetries in $B_d^0\rightarrow J/\psi K_S$ and $B_s^0\rightarrow J/\psi \phi$ [30, 35]^2^

14 $$\begin{aligned} S_{\psi K_S}= 0.691\pm 0.017,\quad S_{\psi \phi }= 0.015\pm 0.035 . \end{aligned}$$

Although $S_{\psi \phi }$ is found to be small it could still significantly differ from its SM value

15 $$\begin{aligned} S_{\psi \phi }^\text {SM}= \sin (2|\beta _s|)=0.036\pm 0.002. \end{aligned}$$

The numerical value for

16 $$\begin{aligned} S_{\psi K_S}^\text {SM}=\sin 2\beta \end{aligned}$$

depends strongly on the value of $|V_{ub}|$, as can be seen from Fig. 4. Here $\beta$ and $\beta _s$ are defined by

17 $$\begin{aligned} V_{td}=|V_{td}|e^{-i\beta }, \quad V_{ts}=-|V_{ts}|e^{-i\beta _s}. \end{aligned}$$

In the LHT model the mass differences $\Delta M_s$ and $\Delta M_d$ are simply given by

18 $$\begin{aligned} \Delta M_s =\frac{G_F^2}{6 \pi ^2}M_W^2 m_{B_s}\left| \lambda _t^{(s)}\right| ^2 F_{B_s}^2\hat{B}_{B_s} \eta _B |S_s| \end{aligned}$$

and

19 $$\begin{aligned} \Delta M_d =\frac{G_F^2}{6 \pi ^2}M_W^2 m_{B_d}\left| \lambda _t^{(d)}\right| ^2 F_{B_d}^2\hat{B}_{B_d} \eta _B |S_d| \end{aligned}$$

with the numerical values of all parameters collected in Table 1.

Next, the presence of new sources of CP violation coming from the T-odd sector modifies the SM formulae in (15), (16) as follows:

20 $$\begin{aligned} S_{\psi K_S} = \sin (2\beta +2\varphi _{B_d}), \quad S_{\psi \phi } = \sin (2|\beta _s|-2\varphi _{B_s}). \end{aligned}$$

Here $\varphi _{B_q}$ are NP phases in $B^0_q$–$\bar{B}^0_q$ mixings. They are directly given in terms of the phases of the loop functions $S_q$:

21 $$\begin{aligned} 2\varphi _{B_q}=-\theta _S^q. \end{aligned}$$

The formulae for $\Delta M_K$ and $\varepsilon _K$ are more complicated because also charm contributions are present. They can all be found in [14]. The only modification relative to these formulae is the change in the overall multiplicative factor in $\varepsilon _K$

22 $$\begin{aligned} e^{i\pi /4} \rightarrow \kappa _\epsilon e^{i\varphi _\epsilon }, \end{aligned}$$

where $\varphi _\epsilon = (43.51\pm 0.05)^\circ$ and $\kappa _\epsilon =0.94\pm 0.02$ [26, 46] takes into account that $\varphi _\epsilon \ne \tfrac{\pi }{4}$ and includes long distance effects in $\mathrm{Im}(\Gamma _{12})$ and $\mathrm{Im}(M_{12})$.

In the following we will present the most interesting branching ratios in terms of the functions $X_i$ and $Y_i$. The CKM elements that we will use are those determined from tree-level decays and consequently they are independent of new physics.

### ${B_{s,d}\rightarrow \mu ^+\mu ^-}$

Interesting implications on the LHT model arise also from the data on $B_{s,d}\rightarrow \mu ^+\mu ^-$. The most recent prediction in the SM that includes NNLO QCD corrections [47] and NLO electroweak corrections [48], put together in [49], and the most recent averages from the combined analysis of CMS and LHCb [50] are given as follows:

23 $$\begin{aligned}&\overline{\mathcal {B}}(B_{s}\rightarrow \mu ^+\mu ^-)_\mathrm{SM} = (3.65\pm 0.23)\cdot 10^{-9},\nonumber \\&\qquad \overline{\mathcal {B}}(B_{s}\rightarrow \mu ^+\mu ^-)_\text {exp} = (2.8^{+0.7}_{-0.6}) \cdot 10^{-9},\end{aligned}$$

24 $$\begin{aligned}&\mathcal {B}(B_{d}\rightarrow \mu ^+\mu ^-)_\mathrm{SM}=(1.06\pm 0.09)\cdot 10^{-10},\nonumber \\&\qquad \mathcal {B}(B_{d}\rightarrow \mu ^+\mu ^-)_\text {exp} =(3.9^{+1.6}_{-1.4})\cdot 10^{-10}. \quad \end{aligned}$$

The “bar” in the case of $B_{s}\rightarrow \mu ^+\mu ^-$ indicates the flavour averaged branching ratio, i. e. $\Delta \Gamma _s$ effects [51–53] have been taken into account in the SM prediction.

As we will be using CKM elements determined in tree-level decays, it is useful to consider the ratios

25 $$\begin{aligned} \mathcal R_s^{\mu \mu }= \frac{\overline{\mathcal {B}}(B_s\rightarrow \mu ^+\mu ^-)}{\overline{\mathcal {B}}(B_s\rightarrow \mu ^+\mu ^-)_\text {SM}}= & {} \left| \frac{Y_s}{Y_\text {SM}}\right| ^2 r(\Delta \Gamma _s), \end{aligned}$$

26 $$\begin{aligned} \mathcal R_d^{\mu \mu }= \frac{\mathcal {B}(B_d\rightarrow \mu ^+\mu ^-)}{\mathcal {B}(B_d\rightarrow \mu ^+\mu ^-)_\text {SM}}= & {} \left| \frac{Y_d}{Y_\text {SM}}\right| ^2, \end{aligned}$$

so that the leading dependence on CKM factors cancels out in these ratios. However, a residual CKM dependence is present in the shifts due to contributions from the T-odd sector, as seen in (11). The factor $r(\Delta \Gamma _s)$ represents the difference between $\Delta \Gamma _s$ effects in the LHT model and in the SM. Using the general formulae in [54] we find in the LHT model

27 $$\begin{aligned} r(\Delta \Gamma _s)=\frac{1+y_s\cos (2\theta ^s_Y-2\varphi _{B_s})}{1+y_s}, \end{aligned}$$

where [35]

28 $$\begin{aligned} y_s=\frac{\Delta \Gamma _s}{2\Gamma _s}=0.061\pm 0.005 . \end{aligned}$$

We find that in the LHT model $r(\Delta \Gamma _s)$ deviates from unity by at most 0.5 % and can therefore be set to unity.

The ratios $\mathcal R_{s,d}^{\mu \mu }$ are independent of the meson weak decay constants. The relevant SM expressions for these branching ratios can be found in [54]. Using these expressions together with (25) and (26) the corresponding results for the LHT model can be found.

While the ratios in question show transparently the size of departures from the SM predictions independently of the values of weak decay constants and CKM parameters, they hide these parametric uncertainties present both in the SM and the LHT model. In particular, both branching ratios depend quadratically on the value of $|V_{cb}|$. The authors in [49] used the inclusive value for $|V_{cb}|\approx 42.2\cdot 10^{-3}$ and obtained the SM result $B_s\rightarrow \mu ^+\mu ^-$ in (23) that is by $1.2\sigma$ above the data. For the exclusive determinations of $|V_{cb}|$, as known presently, the SM would be much closer to the data.

From the point of view of the LHT model it is rather crucial to find out whether the SM prediction is indeed higher than the data or not. Indeed, as we will find in Sect. 5 the LHT model favours a slight enhancement of $\overline{\mathcal {B}}(B_{s}\rightarrow \mu ^+\mu ^-)$ over its SM value, while the data, as seen in (23), favours a moderate suppression. Only a further improvement on the value of $|V_{cb}|$ and the relevant weak decay constants and most importantly future more accurate data can tell whether indeed this is a true problem for the LHT model.

### ${B\rightarrow X_s\gamma }$

The most recent NNLO estimate in the SM gives [55]

29 $$\begin{aligned} \mathcal {B}(B\rightarrow X_s\gamma )_\text {SM} =(3.36\pm 0.23)\cdot 10^{-4}, \end{aligned}$$

which agrees very well with the most recent experimental world average,

30 $$\begin{aligned} \mathcal {B}(B\rightarrow X_s\gamma )_\text {exp} =(3.43\pm 0.22)\cdot 10^{-4}. \end{aligned}$$

The branching ratio for $B\rightarrow X_s\gamma$ decay in the LHT model can be found in [14]. NP effects in this decay turned out to be at the few percent level. Therefore although the room for NP contributions to this decay decreased since 2006, the $B\rightarrow X_s\gamma$ branching ratio still does not pose a relevant constraint, beyond those from $\Delta F=2$ observables, on the LHT parameter space. On the other hand the fact that in this particular case NP effects have been predicted already in 2006 to be small could be regarded as a success of the LHT model. It remains to be seen whether the improvements in the theoretical and experimental accuracy of theory and experiment in this decade will change this picture.

### ${B\rightarrow K^{(*)}\nu \bar{\nu }}$

Of interest are also the exclusive $b\rightarrow s \nu \bar{\nu }$ transitions that are theoretically rather clean and should be measured by Belle II at the end of this decade. The most recent SM estimates of the relevant branching ratios [56] read

31 $$\begin{aligned} \mathcal {B}(B^+\rightarrow K^+\nu \bar{\nu })_\text {SM} = \left[ \frac{|V_{cb}|}{0.0409}\right] ^2(4.0 \pm 0.4) \cdot 10^{-6}, \end{aligned}$$

32 $$\begin{aligned} \mathcal {B}(B^0\rightarrow K^{* 0}\nu \bar{\nu })_\text {SM} = \left[ \frac{|V_{cb}|}{0.0409}\right] ^2 (9.2\pm 0.9) \cdot 10^{-6}, \end{aligned}$$

where the errors in the parentheses are fully dominated by form factor uncertainties. We expect that when these two branching ratios will be measured, these uncertainties will be further decreased and $|V_{cb}|$ will be precisely known so that a very good test of the SM will be possible.

Again the ratios between the LHT and SM predictions for these branching ratios are very simple

33 $$\begin{aligned} \mathcal R^{\nu \nu }_{K}= & {} \frac{\mathcal {B}(B\rightarrow K\nu \bar{\nu })}{\mathcal {B}(B\rightarrow K\nu \bar{\nu })_\text {SM}}= \left| \frac{X_s}{X_\text {SM}}\right| ^2,\end{aligned}$$

34 $$\begin{aligned} \mathcal R^{\nu \nu }_{K^*}= & {} \frac{\mathcal {B}(B\rightarrow K^*\nu \bar{\nu })}{\mathcal {B}(B\rightarrow K^*\nu \bar{\nu })_\text {SM}} = \left| \frac{X_s}{X_\text {SM}}\right| ^2. \end{aligned}$$

Note that similar to models with CMFV these two ratios are equal to each other, which constitutes an important test of the LHT model. This is related to the absence of right-handed flavour changing currents in this model.

### *R*(*D*) and $R(D^*)$

The ratios *R*(*D*) and $R(D^*)$, defined as

35 $$\begin{aligned}&R(D)=\frac{\Gamma (B\rightarrow D\tau \nu )}{\Gamma (B\rightarrow D\ell \nu )},\nonumber \\&R(D^*)=\frac{\Gamma (B\rightarrow D^*\tau \nu )}{\Gamma (B\rightarrow D^*\ell \nu )}, \end{aligned}$$

test the lepton flavour universality in charged current interactions. The recent HFAG average [35] of BaBar [57], Belle [58] and LHCb [59] data

36 $$\begin{aligned}&R(D)_\text {exp}=0.391\pm 0.041 \pm 0.028 ,\nonumber \\&R(D^*)_\text {exp} = 0.322 \pm 0.018 \pm 0.012 \end{aligned}$$

shows a $3.9\sigma$ deviation from the SM prediction [60, 61]

37 $$\begin{aligned} R(D)_\text {SM}=0.297 \pm 0.017,\quad R(D^*)_\text {SM} = 0.252 \pm 0.003. \end{aligned}$$

It is interesting to note that the enhancement with respect to the SM values appears to be universal in both ratios.

Taking a look at the particle content of the LHT model, one might naively hope that this model is able to resolve the anomaly. It has been shown in a model-independent way that a possible solution is the presence of a left-handed charged current contribution [62, 63], mediated by a heavy $W'$ boson. For $f\sim 1\, \mathrm{TeV}$ the new gauge boson $W_H$ is in the right mass range. However, due to T-parity, the new LHT gauge bosons do not couple to SM fermion pairs. Consequently there are no new tree-level contributions to charged current interactions in this model. A new contribution to *R*(*D*) and $R(D^*)$ can arise at the one-loop level, however, the loop suppression together with the smallness of lepton flavour universality breaking effects make it much too small to explain the current *R*(*D*) and $R(D^*)$ anomaly.

### ${K\rightarrow \pi \nu \bar{\nu }}$

The branching ratios for $K^+\rightarrow \pi ^+\nu \bar{\nu }$ and $K_{L}\rightarrow \pi ^0\nu \bar{\nu }$ in the LHT model are given as follows:

38 $$\begin{aligned} \mathcal {B}(K^+\rightarrow \pi ^+\nu \bar{\nu })= & {} \kappa _+ \cdot \left[ \left( \frac{\mathrm{Im}X_\mathrm{eff}}{\lambda ^5}\right) ^2+ \left( \frac{\mathrm{Re}\lambda _c}{\lambda }P_c(X)\right. \right. \nonumber \\&\left. \left. +\frac{\mathrm{Re}X_\mathrm{eff}}{\lambda ^5}\right) ^2\right] , \end{aligned}$$

39 $$\begin{aligned} \mathcal {B}(K_{L}\rightarrow \pi ^0\nu \bar{\nu })=\kappa _L\cdot \left( \frac{\mathrm{Im}X_\mathrm{eff}}{\lambda ^5}\right) ^2, \end{aligned}$$

where [64]

40 $$\begin{aligned} \kappa _+={ (5.173\pm 0.025 )\cdot 10^{-11}\left[ \frac{\lambda }{0.225}\right] ^8}, \end{aligned}$$

41 $$\begin{aligned} \kappa _L= (2.231\pm 0.013)\cdot 10^{-10}\left[ \frac{\lambda }{0.225}\right] ^8 \end{aligned}$$

and $\lambda = |V_{us}|$. For the charm contribution, represented by $P_c(X)$, the calculations in [64–68] imply [69]

42 $$\begin{aligned} P_c(X)= 0.404\pm 0.024, \end{aligned}$$

where the error is dominated by the long-distance uncertainty estimated in [68]. In the following we will assume that NP does not modify this value, which turns out to be true in all known to us extensions of the SM including the LHT model. Such contributions can be in any case absorbed into the function $X_\mathrm{eff}$. The latter function that describes pure short-distance contributions from top quark exchanges and NP contributions in the LHT model is given by

43 $$\begin{aligned} X_\mathrm{eff} = V_{ts}^* V_{td} X_K. \end{aligned}$$

The most recent SM predictions for the branching ratios read [69]

44 $$\begin{aligned}&\mathcal {B}(K^+\rightarrow \pi ^+\nu \bar{\nu })_\text {SM} = \left( 9.11\pm 0.72\right) \cdot 10^{-11},\end{aligned}$$

45 $$\begin{aligned}&\mathcal {B}(K_{L}\rightarrow \pi ^0\nu \bar{\nu })_\text {SM} = \left( 3.00 \pm 0.31\right) \cdot 10^{-11}. \end{aligned}$$

Experimentally we have [70]

46 $$\begin{aligned} \mathcal {B}(K^+\rightarrow \pi ^+\nu \bar{\nu })_\text {exp}=\left( 17.3^{+11.5}_{-10.5}\right) \cdot 10^{-11}, \end{aligned}$$

and the $90\,\%$ C.L. upper bound [71]

47 $$\begin{aligned} \mathcal {B}(K_{L}\rightarrow \pi ^0\nu \bar{\nu })_\text {exp}\le 2.6\cdot 10^{-8}. \end{aligned}$$

Important improvements on these values are expected from the NA62 experiment at CERN in 2018 [72, 73], and from the measurement of $K_{L}\rightarrow \pi ^0\nu \bar{\nu }$ by KOTO around 2020 at J-PARC [74, 75].

### $K_L\rightarrow \mu ^+\mu ^-$

This decay often constrains the size of NP contributions to $K^+\rightarrow \pi ^+\nu \bar{\nu }$. Only the so-called short distance (SD) part to a dispersive contribution to $K_L\rightarrow \mu ^+\mu ^-$ can be reliably calculated. It is given generally as follows ($\lambda =|V_{us}|=0.2252$):

48 $$\begin{aligned} \mathcal {B}(K_L\rightarrow \mu ^+\mu ^-)_\mathrm{SD} = 2.01\cdot 10^{-9} \, \left( \frac{\mathrm{Re} Y^K_\mathrm{eff} }{\lambda ^5} - \bar{P}_c(Y) \right) ^2, \end{aligned}$$

where at NNLO [76]

49 $$\begin{aligned} \bar{P}_c\left( Y\right) \equiv \left( 1-\frac{\lambda ^2}{2}\right) P_c\left( Y\right) ,\quad P_c\left( Y\right) =0.115\pm 0.017. \end{aligned}$$

The SD contributions in the LHT model are described by

50 $$\begin{aligned} Y^K_\mathrm{eff} = V_{ts}^* V_{td} Y_{K} \end{aligned}$$

with

51 $$\begin{aligned} Y_\mathrm{SM} = \eta _Y Y_0(x_t), \quad \eta _Y=0.9982 \end{aligned}$$

also entering the $B_{s,d}\rightarrow \mu ^+\mu ^-$ decays. $Y_0(x_t)$ can be found in [8] and $\eta _Y$ summarizes both QCD and electroweak corrections [49].

As the long-distance contributions to $K_L\rightarrow \mu ^+\mu ^-$ are under poor theoretical control, only a conservative upper bound

52 $$\begin{aligned} \mathcal {B}(K_L\rightarrow \mu ^+\mu ^-)_\mathrm{SD} < 2.5 \cdot 10^{-9} \end{aligned}$$

can be derived [77].

### $\varepsilon '/\varepsilon$

#### SM contribution

The starting point of our presentation is the analytic formula for $\varepsilon '/\varepsilon$ within the SM [39, 78]

53 $$\begin{aligned} \mathrm{{Re}}(\varepsilon '/\varepsilon )_\mathrm{SM}= \mathrm {Im}\lambda _t \cdot F^\text {SM}_{\varepsilon '} \end{aligned}$$

with

54 $$\begin{aligned} F_{\varepsilon '}^\text {SM} =P_0 + P_X \, X_\text {SM} + P_Y \, Y_\text {SM} + P_Z \, Z_\text {SM}+ P_E \, E_\text {SM}~. \end{aligned}$$

The first term in (54) is dominated by QCD-penguin contributions, the next three terms by electroweak penguin contributions and the last term is totally negligible.

Complete information relevant for our analysis can be found in Appendix B of [39]. In particular, the coefficients $P_i$ are given in terms of the non-perturbative parameters

55 $$\begin{aligned}&R_6\equiv B_6^{(1/2)}\left[ \frac{114.54\, \mathrm{MeV}}{m_s(m_c)+m_d(m_c)} \right] ^2,\nonumber \\&\quad R_8 \equiv B_8^{(3/2)}\left[ \frac{114.54\, \mathrm{MeV}}{m_s(m_c)+m_d(m_c)} \right] ^2. \end{aligned}$$

as follows:

56 $$\begin{aligned} P_i = r_i^{(0)} + r_i^{(6)} R_6 + r_i^{(8)} R_8 . \end{aligned}$$

The coefficients $r_i^{(0)}$, $r_i^{(6)}$ and $r_i^{(8)}$ comprise information on the Wilson-coefficient functions of the $\Delta S=1$ weak effective Hamiltonian at the NLO. Their numerical values for three values of $\alpha _s(M_Z)$ are collected in Appendix B of [39].

In our numerical analysis we will use for the quark masses the values [79]

57 $$\begin{aligned}&m_s(2\, \mathrm{GeV})=(93.8\pm 2.4) \, \mathrm{MeV}, \nonumber \\&\quad m_d(2\, \mathrm{GeV})=(4.68\pm 0.16)\, \mathrm{MeV}. \end{aligned}$$

Then at the nominal value $\mu =m_c=1.3\, \mathrm{GeV}$ used in [39], we have

58 $$\begin{aligned}&m_s(m_c)=(109.1\pm 2.8) \, \mathrm{MeV}, \nonumber \\&\quad m_d(m_c)=(5.44\pm 0.19)\, \mathrm{MeV}. \end{aligned}$$

Concerning the parameters $B_6^{(1/2)}$ and $B_8^{(3/2)}$ significant progress has been made since our 2007 analysis [18]. The RBC-UKQCD collaboration [36] determined rather precisely the value of $B_8^{(3/2)}$, which transformed to the NDR scheme and the scale $\mu =m_c$, reads [69]

59 $$\begin{aligned} B_8^{(3/2)}(m_c)=0.76\pm 0.05\, \quad \text {(RBC-UKQCD)} \end{aligned}$$

There is no precise result on $B_6^{(1/2)}$ from lattice QCD. From the most recent results of the RBC-UKQCD collaboration [37] the value of $B_6^{(1/2)}$ has recently been extracted [38, 39]

60 $$\begin{aligned} B_6^{(1/2)}(m_c)=0.57\pm 0.19\, \quad \text {(RBC-UKQCD)}. \end{aligned}$$

But also progress has been made in the large *N* approach of [80] (dual QCD) in which in the large *N* limit one has $B_6^{(1/2)}=B_8^{(3/2)}=1.$ As the recent analysis shows one can derive the bounds [38]

61 $$\begin{aligned} B_6^{(1/2)}\le B_8^{(3/2)}< 1.0. \end{aligned}$$

Moreover, while $B_8^{(3/2)}$ is found in the ballpark of 0.80  $\pm$  0.10, $B_6^{(1/2)}$ is generally smaller and close to the lattice result in (60) but the uncertainties are rather large.

Probably the most important finding of [38] is the bound in (61) which implies an upper bound on $\varepsilon '/\varepsilon$ in the SM. Moreover, it has been shown that the pattern of the size of various matrix elements in this approach is supported by the lattice results in [37].

In a very recent paper [39] a new analysis of $\varepsilon '/\varepsilon$ in the SM has been performed assuming that the $\mathrm{Re}A_0$ and $\mathrm{Re}A_2$ amplitudes are dominated by the SM dynamics. In this manner one could determine the matrix elements of QCD and electroweak penguin $(V-A)\otimes (V-A)$ operators from the precise data on $\mathrm{Re}A_0$ and $\mathrm{Re}A_2$ with much higher precision than it is possible presently from lattice QCD. The outcome of this analysis is the formula for $\varepsilon '/\varepsilon$ in (53) which is given in terms of $B_6^{(1/2)}$ and $B_8^{(3/2)}$.

Using the upper bound in (61), $B_6^{(1/2)}\le B_8^{(3/2)}< 1.0$, one finds, varying all other parameters within their $1\sigma$ ranges [39],

62 $$\begin{aligned} {\mathrm{{Re}}}(\varepsilon '/\varepsilon )_{\mathrm{{SM}}}\le \left[ {\frac{\mathrm{{Im}}\lambda _t}{1.4\cdot 10^{-4}}}\right] \, (8.6\pm 3.2) \cdot 10^{-4}, \end{aligned}$$

roughly by $2\sigma$ below the experimental result [81–84]

63 $$\begin{aligned} \mathrm{{Re}}(\varepsilon '/\varepsilon )_\text {exp}=(16.6\pm 2.3)\cdot 10^{-4}. \end{aligned}$$

Using instead the input from lattice QCD the values for $\varepsilon '/\varepsilon$ in the SM are much lower [39]. We will investigate in Sect. 5 whether the LHT model could help to remove this discrepancy between the theory and data.

#### LHT

The formula for $\varepsilon '/\varepsilon$ in the LHT model reads [18]

64 $$\begin{aligned} {\mathrm{{Re}}(\varepsilon '/\varepsilon )} = |\lambda _t|\, \tilde{F}_{\varepsilon '}, \end{aligned}$$

with

65 $$\begin{aligned} \tilde{F}_{\varepsilon '}= & {} P_0 \sin (\beta -\beta _s) + P_E \, |E_K|\sin \beta _E^K\nonumber \\&+ P_X \, |X_K|\sin \beta _X^K + P_Y \, |Y_K|\sin \beta _Y^K\nonumber \\&+ P_Z \, |Z_K|\sin \beta _Z^K, \end{aligned}$$

where

66 $$\begin{aligned} \beta _i^K = \beta -\beta _s - \theta ^K_i\quad (i = X,Y,Z,E). \end{aligned}$$

The coefficients $P_i$ are the same as in the SM.

### LHT model facing anomalies in $b\rightarrow s \ell ^+\ell ^-$ transitions

The recent highlights in quark flavour physics were the departures of the data on $B_d\rightarrow K^{(*)}\mu ^+\mu ^-$ from the SM expectations, and it is of interest to see how the LHT model faces this data. To this end we recall the shifts caused by NP contributions in the Wilson coefficients $C_9$ and $C_{10}$ of the operators

67 $$\begin{aligned} Q_9 = (\bar{s}\gamma _\mu P_L b)(\bar{\ell }\gamma ^\mu \ell ),\quad Q_{10} = (\bar{s}\gamma _\mu P_L b)(\bar{\ell }\gamma ^\mu \gamma _5\ell ) \end{aligned}$$

in the LHT model. They are

68 $$\begin{aligned} \sin ^2\theta _W C_9^\text {NP}&=\Delta Y_s-4\sin ^2\theta _W \Delta Z_s,\end{aligned}$$

69 $$\begin{aligned} \sin ^2\theta _W C^\text {NP}_{10}&= -\Delta Y_s . \end{aligned}$$

Here,

70 $$\begin{aligned} \Delta Y_s=Y_s-Y_\text {SM},\quad \Delta Z_s= Z_s-Z_\text {SM}. \end{aligned}$$

They can be found by using Eqs. (11) and (12).

The present anomalies in the angular observables in $B_d\rightarrow K^*\mu ^+\mu ^-$ and the suppression of the branching ratio for $B_d\rightarrow K\mu ^+\mu ^-$ below the SM prediction as well as the data on $B_s\rightarrow \mu ^+\mu ^-$ can be well described by [85–88]

71 $$\begin{aligned} C_9^\text {NP}\approx -C^\text {NP}_{10} \approx -(0.5\pm 0.2) . \end{aligned}$$

The solution with NP being present only in $C_9$ is even favoured, but much harder to explain in the context of existing models. We refer to [88] for tables with various solutions and a collection of references to recent papers.

While the anomalies in $B_d\rightarrow K^*\mu ^+\mu ^-$ are subject to theoretical uncertainties, much cleaner is the ratio

72 $$\begin{aligned} \mathcal R^{\mu e}_K= & {} \frac{\mathcal {B}(B^+\rightarrow K^+\mu ^+\mu ^-)^{[1,6]}}{\mathcal {B}(B^+\rightarrow K^+ e^+ e^-)^{[1,6]}}\nonumber \\= & {} 0.745^{+0.090}_{-0.074}(\text {stat})\pm 0.036(\text {syst}) , \end{aligned}$$

where the quoted value is the one from LHCb [89]. It is by $2.6\sigma$ lower than its SM value $1 +\mathcal {O}(10^{-4})$ and is an intriguing signal of the breakdown of lepton flavour universality.

All these anomalies turn out to be a problem for the LHT model. The relation (71) is badly violated in the LHT model, where due to the smallness of the muon vector coupling in the *Z* penguin $C_9^\text {NP}$ turns out to be by an order of magnitude smaller than $C_{10}^\text {NP}$. Moreover, $C_{10}^\text {NP}<0$, in variance with (71), is favoured in the LHT model. This is the origin of the enhancement of $B_s\rightarrow \mu ^+\mu ^-$ in this model mentioned above. In addition the breakdown of lepton universality in the LHT model is absent at the tree-level and even if it can be generated at one-loop level, it is by far too small to explain the result in (72).

Thus, these anomalies, if confirmed by future more accurate data, have the power to exclude the LHT model as the source of the observed pattern of departures from SM expectations for $b\rightarrow s \ell ^+\ell ^-$ transitions.

### $D^0$–$\bar{D}^0$ mixing

LHT contributions to $D^0$–$\bar{D}^0$ mixing and CP violation have been investigated in detail in [17, 20]. In the present paper we refrain from repeating this analysis, however, we would like to briefly comment on how the situation changed since 2009.

As in 2009, $D^0$–$\bar{D}^0$ mixing in the SM is still plagued by significant hadronic uncertainties. The latter prevent us from obtaining clean correlations between *K* and *D* meson observables in the LHT model, which, a priori, are expected in models with only left-handed currents [90]. The improved experimental constraints on CP violation in $D^0$–$\bar{D}^0$ mixing [35] therefore do not have a relevant impact on our results for *K* and $B_{d,s}$ physics observables, which we also confirmed numerically.

## Constraints on the LHT parameter space

The previous two sections summarized the expressions for flavour observables to be used in our numerical analysis. But, in addition, experiments from the various areas of particle physics place strong constraints on the parameter space of the LHT model and they have to be taken into account. While the indirect constraints from electroweak precision (EWP) physics are largely unchanged with respect to our earlier analyses, major improvements have been achieved on direct bounds thanks to the first LHC run. Additionally, the discovery of the Higgs boson and the measurement of its mass as well as its production and decay rates yields new and partly complementary input. A major analysis of current constraints on the LHT parameter space has been presented in [34]. In the following we briefly recapitulate the new LHT parameters relevant for our analysis and review the current constraints.

### Electroweak and top sector

In the electroweak sector the only new parameter is the scale *f* at which the $SU(5)\rightarrow SO(5)$ global symmetry breaking takes place. It determines the mass of the new heavy gauge bosons and scalars and sets the mass scale for the new fermions.

In the top sector the parameter $x_L$ describes the mixing between the top quark and its T-even partner $T_+$. It also determines the masses of the $T_+$ and $T_-$ quarks, the latter of which is not relevant for FCNC processes. These parameters are most stringently constrained indirectly, namely from EWP and Higgs data.

EWP constraints on the LHT model have been studied in detail in [91], and in the context of a simplified model in [92]. Recently these analyses have been updated in [34], including the measured value of the Higgs mass $m_h \sim 125\, \mathrm{GeV}$ as well as the T-odd fermion contributions. Interestingly the performed $\chi ^2$ fit showed that scales as low as $\sim$400$\, \mathrm{GeV}$ are still consistent with EWP data if the parameter $x_L$, describing the mixing between the top quark and its partner $T_+$, is close to 0.5.

The bound on the symmetry breaking scale *f*, however, increases significantly when the LHC Higgs data are taken into account. Higgs searches alone constrain the scale *f* to be above $\sim$600$\, \mathrm{GeV}$, independently of the parameter $x_L$ [34].

Combining electroweak and Higgs physics constraints yields the lower bound [34]

73 $$\begin{aligned} f\mathop {_\sim }\limits ^{>}694\, \mathrm{GeV}\quad \text {at 95\,\% C.L.} \end{aligned}$$

with $x_L\simeq 0.5$. This corresponds to a fine-tuning of at least 5 %.

Interestingly the choice

74 $$\begin{aligned} f = 1\, \mathrm{TeV},\quad x_L = 0.5 \end{aligned}$$

we had made in our earlier analyses [14, 15, 21] is still consistent with the currently available indirect constraints. Note that this choice fixes

75 $$\begin{aligned} m_{T_+} = 1.4\, \mathrm{TeV}\end{aligned}$$

which is still well beyond direct limits from the LHC.

### Mirror quark sector

The majority of new parameters in the LHT model is intimately tied to the flavour sector. They arise from the mass matrices of mirror quarks and leptons. Only the mass matrix for mirror quarks is relevant in the present paper. It introduces nine new parameters that can be conveniently divided into the three masses^3^ and a flavour mixing matrix $V_{Hd}$ with three angles and three CP-violating phases. These are

76 $$\begin{aligned} m^q_{H1},\quad m^q_{H2},\quad m^q_{H3},\quad \theta _{12}^d,\quad \theta _{13}^d,\quad \theta _{23}^d,\quad \delta _{12}^d,\quad \delta _{13}^d,\quad \delta _{23}^d, \end{aligned}$$

where the last six parametrise the matrix $V_{Hd}$ in terms of the parametrisation presented in [44].

#### Bounds on mirror quark masses

The most stringent bounds on the LHT mass spectrum from the LHC experiments are on the mirror quarks, due to their strong coupling to quarks and gluons. Similarly to squarks in supersymmetry, they are pair produced by strong interactions and lead to missing energy signatures with jets and possibly leptons in the final state.

In an early analysis [93] the CMS search for jets and missing transverse energy was used to derive the expected bound $m^q_{H} \mathop {_\sim }\limits ^{>}650\, \mathrm{GeV}$ for $1\,\text {fb}^{-1}$ of data at $\sqrt{s}=7\, \mathrm{TeV}$. By now a significantly higher integrated luminosity is available, and many squark searches with different final states have been presented by ATLAS and CMS. The searches most sensitive to LHT mirror quarks have been recast in [34]. Interestingly the most stringent constraints have been found to arise from the search for jets, leptons and missing energy, since the mirror quarks dominantly decay into the heavy gauge bosons $W_H^\pm$, $Z_H$ subsequently producing final state leptons. Assuming a degenerate mirror quark spectrum, for $f=1\, \mathrm{TeV}$ the lower bound

77 $$\begin{aligned} m^q_{H}\mathop {_\sim }\limits ^{>}1600\, \mathrm{GeV}\end{aligned}$$

has been obtained.

It should be stressed that the bounds on individual mirror quarks can be weaker if the requirement of degeneracy is lifted, similarly to the case of non-degenerate squarks [94]. Furthermore the presence of flavour mixing between the various generations affects the constraints [95].

Upper bounds on the mirror fermion masses can be obtained from their non-decoupling contribution to four-fermion operators [91]. The constraint on the mirror fermion masses scales linearly with the scale *f*. For $f=1\, \mathrm{TeV}$, and assuming degenerate mirror fermions, the current bound from LEP and LHC data [34] is roughly

78 $$\begin{aligned} m_{H}\mathop {_\sim }\limits ^{<}4.6\, \mathrm{TeV}. \end{aligned}$$

#### Constraints on mixing parameters

In contrast to the other LHT parameters, the parameters of the mixing matrix $V_{Hd}$ cannot be constrained by determining the mass spectrum of new particles. However, they will in principle be accessible to the LHC by measuring the decays of the mirror quarks into the various SM flavours. Such measurements of branching ratios and CP-asymmetries would indeed allow for the most direct determination of mixing angles and CP-violating phases in the mirror sector. Similarly to the determination of the CKM matrix from tree level decays, such a method gives the most direct access to the parameters in question.

This task will, however, be challenging if not impossible to accomplish at the LHC. Luckily FCNC processes come to the rescue here. Even with their help the determination of all these flavour mixing parameters is clearly a very difficult task, in particular if no LHT particles will be discovered at the LHC. On the other hand if in the second round of LHC operation new particles present in the LHT model will be discovered, we will be able to determine *f* from $M_{W_H}$, $M_{Z_H}$ or $M_{A_H}$ and $x_L$ from $m_{T_-}$ or $m_{T_+}$. Similarly the mirror fermion masses $m_{Hi}$ will be measured.

Since the CKM parameters can be determined independently of the LHT contributions from tree-level decays during the flavour precision era, the only remaining free parameters in the quark sector are $\theta _{ij}^d$ and $\delta _{ij}^d$. They can, similarly to the parameters of the CKM matrix, be determined with the help of loop induced flavour-violating processes. How this determination of the matrix $V_{Hd}$ from loop induced decays would be realised in practice has already been discussed in [14, 15] and we will not repeat it here.

### Parameter choices for our analysis

In our analysis we will study two different scenarios for the LHT mass scales.

**Scenario A** The first one assumes a low new physics scale

79 $$\begin{aligned} f=1\, \mathrm{TeV}, \end{aligned}$$

in the reach of the LHC. A low value is clearly preferred by naturalness arguments. In order to optimise the agreement with EWP data, as in our earlier analyses we set the mixing parameter

80 $$\begin{aligned} x_L = 0.5. \end{aligned}$$

The mirror quark masses will be varied in the range $(i=1,2,3)$

81 $$\begin{aligned} 1600\, \mathrm{GeV}< m^q_{Hi} < 4500\, \mathrm{GeV}\end{aligned}$$

in agreement with the current constraints.

In this context we recall that the T-odd contributions to FCNC processes are governed by the exchange of mirror fermions and the new gauge bosons in loop diagrams. Consequently the mass splittings between mirror fermions belonging to different doublets are strongly bounded by FCNC processes in correlation with the departure of the matrix $V_{Hd}$ from the unit matrix.

**Scenario B** The second scenario studies the pessimistic case that no new particles will be found at the LHC in the coming years and no clear deviations from the SM predictions for EWP observables will be found. Our goal here will then be to find how large deviations from SM predictions will still be allowed, with the hope that some deviations from SM predictions in FCNC observables will be detected. Lacking a detailed analysis of the LHC reach, clearly we can only guess what the bounds on the LHT scales will then be.

The improved knowledge of EWP and Higgs observables will push the symmetry breaking scale *f* up to several TeV. We choose

82 $$\begin{aligned} f=3\, \mathrm{TeV},\quad x_L = 0.5 \end{aligned}$$

as a benchmark value. Again the latter choice minimizes the LHT contributions to EWP observables.

The direct bounds on mirror quarks will push their masses in the multi-TeV regime, and we choose

83 $$\begin{aligned} 4 \, \mathrm{TeV}< m^q_H < 8 \, \mathrm{TeV}, \end{aligned}$$

with the upper bound obtained from an expected improvement on the four-fermion operator constraints.

Before proceeding to the numerical analysis, we note that the LHT model suffers from severe fine-tuning in this case. This questions the original motivation for Little Higgs models as a natural solution to the little hierarchy problem. However, we still think that a high scale scenario is worth being considered in terms of its flavour phenomenology. In the absence of a new physics discovery at the LHC, most new physics scenarios will have a severe fine-tuning problem and the naturalness hypothesis will be challenged. In this case it will be important to question the concept of naturalness as one of our main guiding principles. No stone should be left unturned in the search for new physics, even if a model seems theoretically less motivated. In this spirit we consider it worth investigating whether in the absence of a NP signal in direct searches and Higgs data, flavour-violating decays can still show a significant deviation from the SM prediction.

## Numerical analysis 

### Strategy

An important part of our analysis is the choice of the values of CKM parameters as this specifies the room left for NP contributions. We will use the CKM parameters determined in tree-level decays. These are

84 $$\begin{aligned} |V_{us}|, \quad |V_{ub}|, \quad |V_{cb}|, \quad \gamma . \end{aligned}$$

The values for $|V_{us}|$ and the angle $\gamma$ used by us are [35, 102]

85 $$\begin{aligned} |V_{us}| = 0.2253\pm 0.0008,\quad \gamma = (73.2^{+6.3}_{-7.0})^\circ . \end{aligned}$$

The status of $|V_{cb}|$ is not satisfactory, with exclusive determinations [79, 103, 104] giving significantly lower values than the inclusive [105] ones

86 $$\begin{aligned}&|V_{cb}|_\text {excl}=(39.36\pm 0.75)\cdot 10^{-3},\nonumber \\&\quad |V_{cb}|_\text {incl}=(42.21\pm 0.78)\cdot 10^{-3}, \end{aligned}$$

implying the weighted average of these results provided in [69]

87 $$\begin{aligned} |V_{cb}|_\mathrm{avg}=(40.7\pm 1.4)\cdot 10^{-3} \end{aligned}$$

that we will adopt in the following.

The status of $|V_{ub}|$ is even worse due to the tensions between exclusive [104] and inclusive [79] determinations of $|V_{ub}|$:

88 $$\begin{aligned}&|V_{ub}|_\text {excl}=(3.72\pm 0.14)\cdot 10^{-3},\nonumber \\&\quad |V_{ub}|_\text {incl}=(4.40\pm 0.25)\cdot 10^{-3}. \end{aligned}$$

The weighted average of these results provided in [69] reads

89 $$\begin{aligned} |V_{ub}|_\mathrm{avg} =(3.88\pm 0.29)\cdot 10^{-3}, \end{aligned}$$

but due to the recent LHCb result which gives the even lower value of $|V_{ub}|=3.25\cdot 10^{-3}$ the situation is rather unclear. For the time being we will use the value in (89).

In this context, it should be mentioned that, using the central values of other input parameters, even with the inclusive value of $|V_{cb}|$, the value of $\varepsilon _K$ in the SM is typically by $(10{-}20)\,\%$ below the data, unless the high inclusive value of $|V_{ub}|$ is used. However, the large uncertainty in $\eta _{cc}$ found at NNLO level in [100] implies an uncertainty of roughly $\pm 6\,\%$ in $\varepsilon _K$ softening the tension in the SM with $\varepsilon _K$. For a recent discussion see [106].

On the other hand for $|V_{ub}|\ge 3.6\cdot 10^{-3}$ the asymmetry $S_{\psi K_S}$ predicted by the SM is larger than its experimental value. For the inclusive value of $|V_{ub}|$ it is even by $3\sigma$ above the data. Then new CP phases in the $B_d^0-\bar{B}_d^0$ system are required to achieve an agreement with experiment, while then $\varepsilon _K$ in the SM is fully consistent with the data. Thus some tension between the values of $\varepsilon _K$ and $S_{\psi K_S}$ in the SM is still present [25, 26], but to reach a final conclusion, much higher accuracies on $|V_{ub}|$, $|V_{cb}|$ and also on $\eta _{cc}$ are required.

The remaining input parameters are collected in Table 1. We will comment on some of them whenever necessary. For the new parameters of the LHT model we will impose the bounds summarised in Sect. 4. As in our 2009 analysis [21] we perform a randomised numerical scan over the LHT parameter space, varying the input parameters in their $1\sigma$ ranges. For both scenarios A and B we generate a set of 10,000 parameter points each that satisfy the present $\Delta F = 2$ constraints at the $1\sigma$ level.

### Results for scenario A

#### $\Delta F=2$ constraints

The presence of new contributions to the $\Delta F =2$ observables in (13) and (14) allows one to resolve possible tensions present in the SM, thereby putting some constraints on the new parameters. These $\Delta F=2$ constraints will be taken into account in the predictions for $\Delta F=1$ observables presented below.

**Fig. 1:**
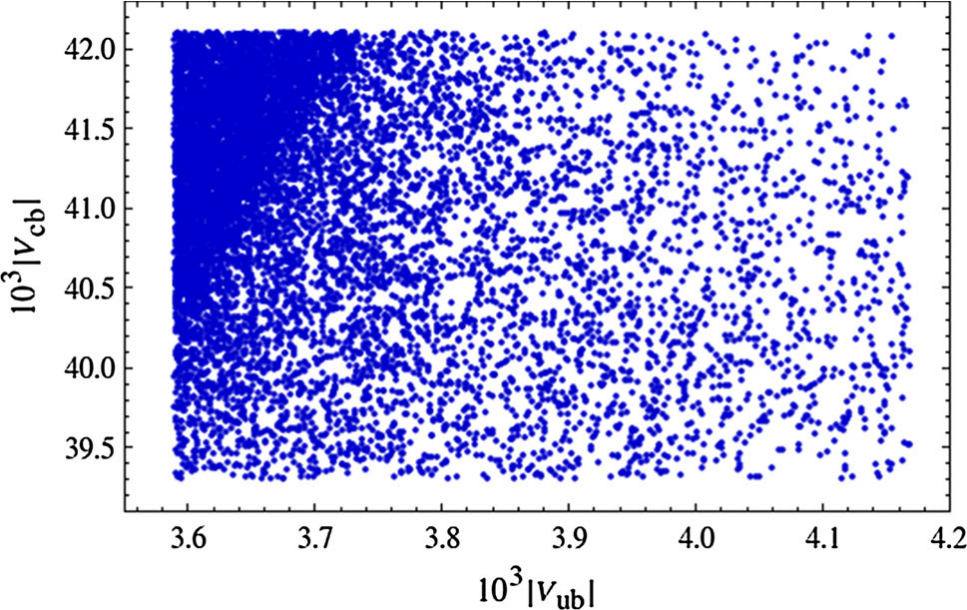
Distribution of viable LHT parameter points ($f=1\, \mathrm{TeV}$) in the $|V_{ub}|,|V_{cb}|$ plane, obtained with a prior flat distribution

At this point it should be recalled that in the LHT model the CP asymmetry $S_{\psi \phi }$ can both be enhanced and suppressed w. r. t. the SM. We will see this in the figures below. This is not always the case in other models. For instance in the Two Higgs Doublet Model with MFV and flavour blind phases ($\mathrm{2HDM_{\overline{MFV}}}$) [31, 32], the asymmetry $S_{\psi \phi }$ can only be enhanced due to its correlation with $S_{\psi K_S}$. Thus if eventually $S_{\psi \phi } < (S_{\psi \phi })_\text {SM}$ will be found, the LHT model will still be viable, in contrast to the $\mathrm{2HDM_{\overline{MFV}}}$.

Figure 1 demonstrates that the LHT model can fit the data on $\Delta F=2$ observables for the full range of the measured values of $|V_{ub}|$ and $|V_{cb}|$ covered in our scan. Yet, small values of $|V_{ub}|$ and large values of $|V_{cb}|$ are favoured as for such values the data on $S_{\psi K_S}$ and $\varepsilon _K$ are easiest to satisfy, respectively.

**Fig. 2:**
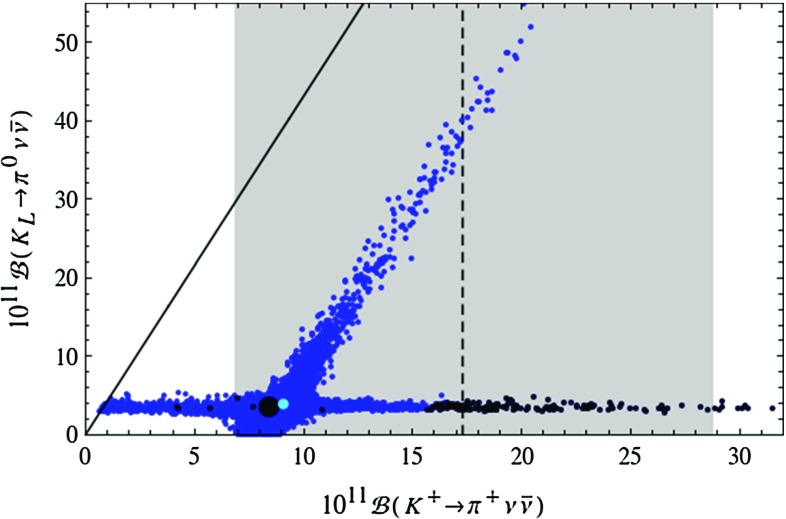
Correlation between the branching ratios of $K^+\rightarrow \pi ^+\nu \bar{\nu }$ and $K_L\rightarrow \pi ^0\nu \bar{\nu }$ in the LHT model for $f=1\, \mathrm{TeV}$. The *large black dot* shows the central SM value for our choice of input parameters, and the *light blue point* shows the contribution from the T-even sector. The *black LHT points* are excluded by the constraint from $K_L\rightarrow \mu ^+\mu ^-$ [77]. The experimental $1\sigma$ range for $\mathcal {B}(K^+\rightarrow \pi ^+\nu \bar{\nu })$ [70] is displayed by the *grey band*, while the *solid black line* indicates the Grossman–Nir bound [107]

#### $K^+\rightarrow \pi ^+\nu \bar{\nu }$ and $K_{L}\rightarrow \pi ^0\nu \bar{\nu }$

The correlation between $K^+\rightarrow \pi ^+\nu \bar{\nu }$ and $K_{L}\rightarrow \pi ^0\nu \bar{\nu }$ has been the subject of many analyses. In Fig. 2 we show the correlation between $\mathcal {B}(K^+\rightarrow \pi ^+\nu \bar{\nu })$ and $\mathcal {B}(K_{L}\rightarrow \pi ^0\nu \bar{\nu })$ as obtained from the randomised scan over the LHT parameters. The experimental $1\sigma$ range for $\mathcal {B}(K^+\rightarrow \pi ^+\nu \bar{\nu })$ [70] and the model-independent Grossman–Nir (GN) bound [107] are also shown. We observe that the two branches of possible points found in [15] are still present and that significant enhancements with respect to the SM predictions are allowed. In fact the possible enhancements are larger than in our 2009 analysis. This counter-intuitive result originates in the non-decoupling behaviour of the mirror quarks which, due to the constraints from LHC Run 1, have to be heavier than assumed by us 6 years ago. The first branch, which is parallel to the GN-bound, leads to possible large enhancements in $\mathcal {B}(K_{L}\rightarrow \pi ^0\nu \bar{\nu })$ so that, without the constraint from $\varepsilon '/\varepsilon$, values as high as $5\cdot 10^{-10}$ are possible, being at the same time consistent with the measured value for $\mathcal {B}(K^+\rightarrow \pi ^+\nu \bar{\nu })$. The latter branching ratio can reach values in the ballpark of $2\cdot 10^{-10}$. On the second branch, which corresponds to values for $\mathcal {B}(K_{L}\rightarrow \pi ^0\nu \bar{\nu })$ rather close to its SM prediction, $\mathcal {B}(K^+\rightarrow \pi ^+\nu \bar{\nu })$ can be strongly suppressed but also enhanced. However, the size of this enhancement is limited by the $K_L\rightarrow \mu ^+\mu ^-$ constraint so that the present central experimental value can only barely be reached. We will return to this constraint in explicit terms below.

The presence of the two branches is a remnant of the specific operator structure of the LHT model and has been analysed in a model-independent manner in [33]. Consequently observing one day the $K\rightarrow \pi \nu \bar{\nu }$ branching ratios outside these two branches would not only rule out the LHT model but at the same time put all models with a similar flavour structure in difficulties. On the other hand in models like the custodially protected Randall–Sundrum (RS) model in which new flavour-violating operators are present, no visible correlation is observed, so that an observation of the $K\rightarrow \pi \nu \bar{\nu }$ modes outside the two branches can be explained in such kind of models [108]. This is also possible in models with tree-level flavour-violating *Z* and $Z^\prime$ exchanges [109, 110] if flavour changing left- and right-handed couplings are present.

**Fig. 3:**
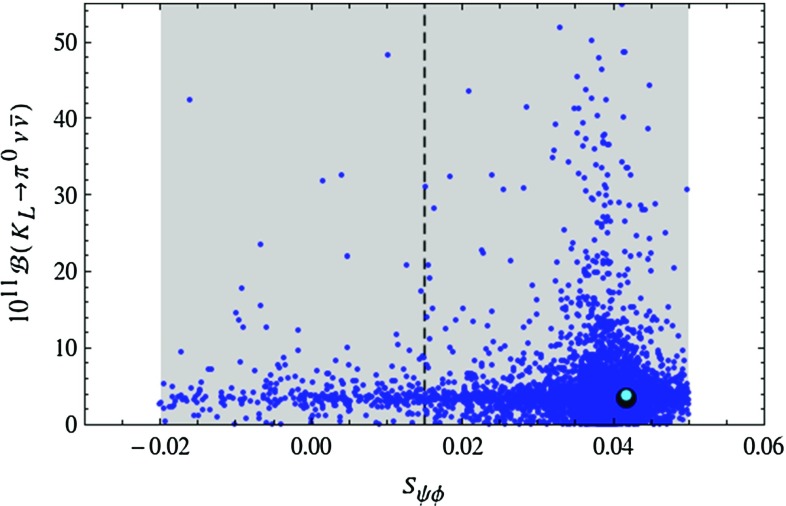
Branching ratio of $K_L\rightarrow \pi ^0\nu \bar{\nu }$ as a function of $S_{\psi \phi }$ in the LHT model for $f=1\, \mathrm{TeV}$. The *large black dot* shows the central SM value for our choice of input parameters, and the *light blue point* shows the contribution from the T-even sector. The experimental $1\sigma$ range for $S_{\psi \phi }$ is displayed by the *grey band* [35]

#### $K_{L}\rightarrow \pi ^0\nu \bar{\nu }$, $S_{\psi K_S}$ and $S_{\psi \phi }$.

Next, of particular interest are the correlations of $K_{L}\rightarrow \pi ^0\nu \bar{\nu }$ with the asymmetries $S_{\psi K_S}$ and $S_{\psi \phi }$. In 2009 we have pointed out that large departures of $S_{\psi \phi }$ from its SM value would not allow for large NP effects in the rare *K* decay within the LHT model. But as seen in (14) the present experimental value for this asymmetry fully agrees with the SM. In Fig. 3 we show the correlation of $\mathcal {B}(K_{L}\rightarrow \pi ^0\nu \bar{\nu })$ with $S_{\psi \phi }$. We observe that within the LHT model $S_{\psi \phi }$ can still differ significantly from its SM value of 0.04, but large enhancements of $\mathcal {B}(K_{L}\rightarrow \pi ^0\nu \bar{\nu })$ are most likely when $S_{\psi \phi }$ is SM-like. It should also be noted that the large new physics effects are due to mirror fermions as the T-even sector is CMFV like.

**Fig. 4:**
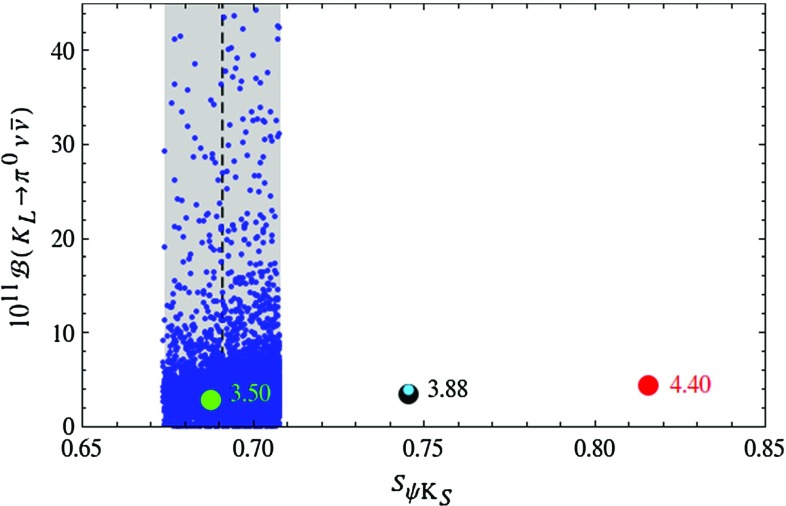
Branching ratio of $K_L\rightarrow \pi ^0\nu \bar{\nu }$ as a function of $S_{\psi K_S}$ in the LHT model for $f=1\, \mathrm{TeV}$. The *large black dot* shows the central SM value for our choice of input parameters, and the *light blue point* shows the contribution from the T-even sector. The *green* and *red dots* indicate the SM predictions for $|V_{ub}|_\text {excl.}=3.5\cdot 10^{-3}$ and $|V_{ub}|_\text {incl.}=4.4\cdot 10^{-3}$, respectively. The experimental $1\sigma$ range for $S_{\psi K_S}$ is displayed by the grey band [35]

Figure 4 demonstrates that for high values of $|V_{ub}|$ the T-even sector would not be capable to lower the value of $S_{\psi K_S}$ to agree with the data, while this can be achieved with the help of the mirror fermions simultaneously allowing for significant departures of the branching ratio for $K_{L}\rightarrow \pi ^0\nu \bar{\nu }$ from its SM value.

#### Correlation of $K\rightarrow \pi \nu \bar{\nu }$ with $K_L\rightarrow \mu ^+\mu ^-$ and $\varepsilon '/\varepsilon$

**Fig. 5:**
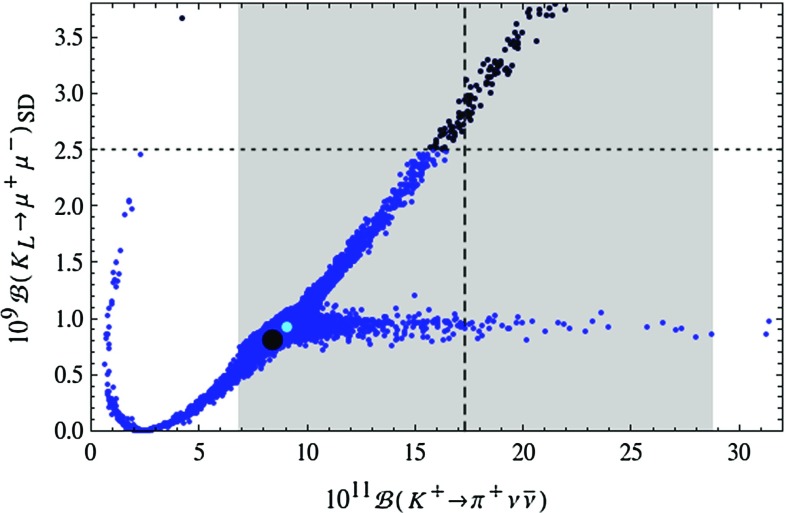
Correlation between the short-distance contribution to $\mathcal {B}(K_L\rightarrow \mu ^+\mu ^-)$ and the branching ratio of $K^+\rightarrow \pi ^+\nu \bar{\nu }$ in the LHT model for $f=1\, \mathrm{TeV}$. The *large black dot* shows the central SM value for our choice of input parameters, and the *light blue point* shows the contribution from the T-even sector. The *black LHT points* are excluded by the constraint from $K_L\rightarrow \mu ^+\mu ^-$, indicated by the *horizontal dotted line* [77]. The experimental $1\sigma$ range for $\mathcal {B}(K^+\rightarrow \pi ^+\nu \bar{\nu })$ is displayed by the *grey band* [70]

Of interest are also the correlations of $K\rightarrow \pi \nu \bar{\nu }$ with $K_L\rightarrow \mu ^+\mu ^-$ and $\varepsilon '/\varepsilon$ as they can limit possible NP effects in $K\rightarrow \pi \nu \bar{\nu }$. In Fig. 5 we show the correlation between $K_L \rightarrow \mu ^+ \mu ^-$ and $K^+\rightarrow \pi ^+\nu \bar{\nu }$. As pointed out in [108] this linear correlation on the upper branch should be contrasted with the inverse correlation between the two decays in question found in the custodially protected RS model. The origin of this difference is the operator structure of the models in question: while in the LHT model rare *K* decays are mediated as in the SM by left-handed currents, in the RS model in question the flavour-violating *Z* coupling to right-handed quarks dominates. In the LHT model consequently a large enhancement of $\mathcal {B}(K^+\rightarrow \pi ^+\nu \bar{\nu })$ automatically implies a significant enhancement of $\mathcal {B}(K_L \rightarrow \mu ^+ \mu ^-)_\text {SD}$ and this is not always allowed by the upper bound $\mathcal {B}(K_L \rightarrow \mu ^+ \mu ^-)_\text {SD}<2.5 \cdot 10^{-9}$ [77], displayed by the dotted line in Fig. 5. The horizontal branch in this figure, on which $\mathcal {B}(K^+\rightarrow \pi ^+\nu \bar{\nu })$ is not constrained by $K_L\rightarrow \mu ^+\mu ^-$, corresponds to the upper branch in Fig. 2, while the upper one in Fig. 5 to the lower one in Fig. 2.

Another interesting correlation is the one of $K_{L}\rightarrow \pi ^0\nu \bar{\nu }$ and $\varepsilon '/\varepsilon$ which has been analysed by us in the LHT model in [18]. As we summarised in Sect. 3.8 significant progress has been made since then both by lattice QCD and large *N* through the improved determination of the relevant hadronic matrix elements of QCD and electroweak penguin operators. Using the upper bound on $B_6^{(1/2)}$ and $B_8^{(3/2)}$ in (61) the authors of [39] find $\varepsilon '/\varepsilon$ in the SM at the bound in (62) to be roughly by $2\sigma$ lower than the data.

**Fig. 6:**
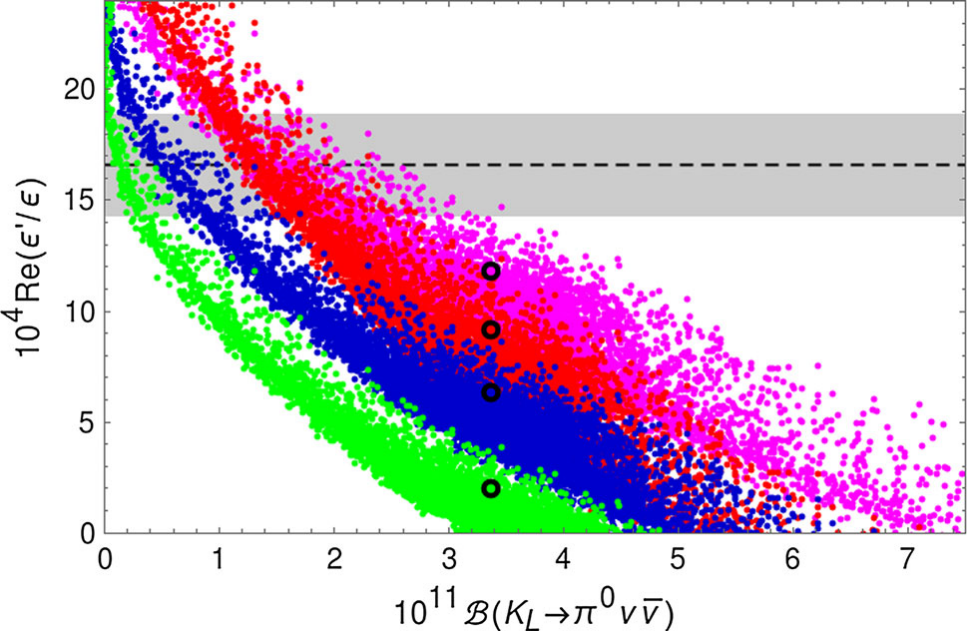
Correlation between $\mathcal {B}(K_L\rightarrow \pi ^0\nu \bar{\nu })$ and $\mathrm{{Re}}(\varepsilon '/\varepsilon )$ in the LHT model for $f=1\, \mathrm{TeV}$ for different values of $(B_6^{(1/2)},B_8^{(3/2)})$: (1.0, 1.0) (*red*), (0.76, 0.76) (*blue*), (0.57, 0.76) (*green*), (1.0, 0.76) (*magenta*). The *black dots* show the corresponding central SM values. The experimental $1\sigma$ range for $\mathrm{{Re}}(\varepsilon '/\varepsilon )$ is displayed by the *grey band* [81–84]

In our analysis we will consider first of all three choices for the pair $(B_6^{(1/2)},B_8^{(3/2)})$:

90 $$\begin{aligned} B_6^{(1/2)}=B_8^{(3/2)}=1.0, \quad (\text {red}), \end{aligned}$$

corresponding to the upper bound in (61),

91 $$\begin{aligned} B_6^{(1/2)}=B_8^{(3/2)}= 0.76, \quad (\text {blue}), \end{aligned}$$

corresponding to the central lattice value for $B_8^{(3/2)}$ and the largest value for $B_6^{(1/2)}$ consistent with the bound in (61) and

92 $$\begin{aligned} B_6^{(1/2)}=0.57, \quad B_8^{(3/2)}=0.76 \quad (\text {green}) \end{aligned}$$

corresponding to the central lattice values.

In Fig. 6 we show the correlation between $K_{L}\rightarrow \pi ^0\nu \bar{\nu }$ and $\varepsilon '/\varepsilon$ for these three scenarios. We observe that in the second and third case the SM prediction is significantly below the data. Requiring the LHT model to obtain agreement with the data suppresses strongly the branching ratio $\mathcal {B}(K_L\rightarrow \pi ^0\nu \bar{\nu })$ below its SM value. At the bound in (90) taking all the uncertainties into account the suppression is moderate. This is in particular the case if we allow one to violate the inequality between $B_6^{(1/2)}$ and $B_8^{(3/2)}$ and choose

93 $$\begin{aligned} B_6^{(1/2)}=1.0, \quad B_8^{(3/2)}=0.76 \quad (\text {magenta}). \end{aligned}$$

But this case is very unlikely in view of the bound in (61).

**Fig. 7:**
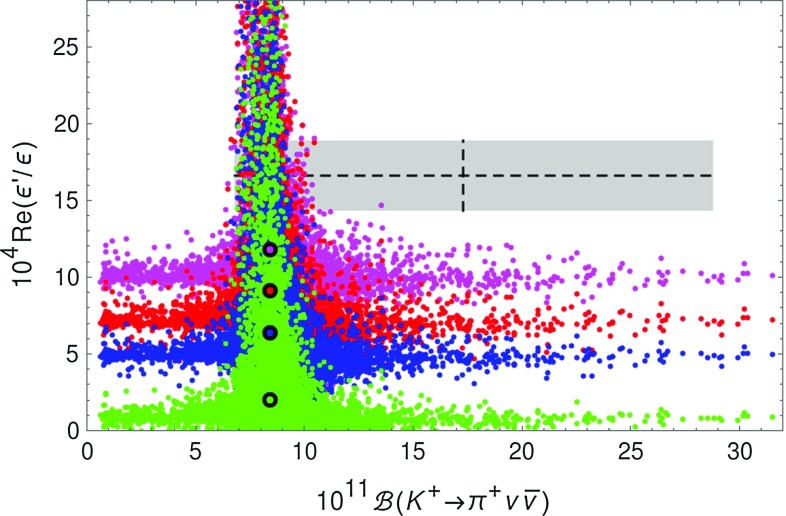
Correlation between $\mathcal {B}(K^+\rightarrow \pi ^+\nu \bar{\nu })$ and $\mathrm{{Re}}(\varepsilon '/\varepsilon )$ in the LHT model for $f=1\, \mathrm{TeV}$ for different values of $(B_6^{(1/2)},B_8^{(3/2)})$: (1.0, 1.0) (*red*), (0.76, 0.76) (*blue*), (0.57, 0.76) (*green*), (1.0, 0.76) (*magenta*). The *black dots* show the corresponding central SM values. The experimental $1\sigma$ ranges are displayed by the *grey band* [70, 81–84]

Figure 7 shows the analogous correlation between $\mathcal {B}(K^+\rightarrow \pi ^+\nu \bar{\nu })$ and $\mathrm{{Re}}(\varepsilon '/\varepsilon )$. The two branches of Fig. 2 also manifest themselves in the present figure. The horizontal branch with large enhancements of $\mathcal {B}(K^+\rightarrow \pi ^+\nu \bar{\nu })$ is disfavoured by $\varepsilon '/\varepsilon$. Fitting the data on $\varepsilon '/\varepsilon$ is possible within the LHT model without any suppression of $\mathcal {B}(K^+\rightarrow \pi ^+\nu \bar{\nu })$. However, significant modifications of this branching ratio with respect to the SM are then not allowed.

#### Problems with $B_{s,d}\rightarrow \mu ^+\mu ^-$ and $B_d\rightarrow K^{(*)} \ell ^+\ell ^-$

While until now the LHT model passed all experimental tests related to $\Delta F=2$ transitions and rare *K* decays, the situation changes when $B_{s,d}\rightarrow \mu ^+\mu ^-$ and $B_d\rightarrow K^{(*)} \ell ^+\ell ^-$ are considered.

**Fig. 8:**
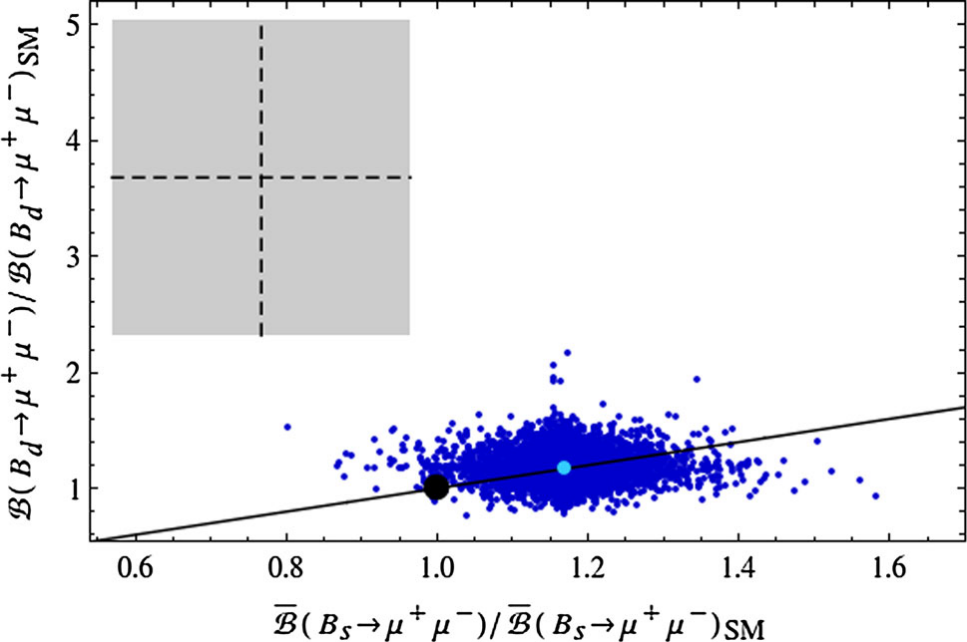
Correlation between $\bar{\mathcal {B}}(B_s\rightarrow \mu ^+\mu ^-)$ and $\mathcal {B}(B_d\rightarrow \mu ^+\mu ^-)$ in the LHT model for $f=1\, \mathrm{TeV}$. The *large black dot* shows the central SM value for our choice of input parameters, and the *light blue point* shows the contribution from the T-even sector. The experimental $1\sigma$ ranges are displayed by the *grey rectangle* [50], and the MFV prediction is indicated by the *solid black line*

In Fig. 8 we show the correlation between the ratios $\mathcal R_{s,d}^{\mu \mu }$ in the LHT model. While the MFV prediction, represented by the straight black line, can be modified, this modification is not sufficient to bring the theory in full agreement with the data. While the data would favour a suppression of $\mathcal {B}(B_s\rightarrow \mu ^+\mu ^-)$ relative to its SM value, the LHT model favours its enhancement. The contribution from the T-even sector provides a flavour universal enhancement by $15\,\%$, and particular values of model parameters in the T-odd sector are required to change this pattern. We find that while the mirror quarks can enhance $\mathcal {B}(B_d\rightarrow \mu ^+\mu ^-)$ by up to a factor of 2, such large values appear impossible together with a suppression of $\mathcal {B}(B_s\rightarrow \mu ^+\mu ^-)$. Consequently finding future data to confirm the present ranges of $\mathcal {B}(B_{s,d}\rightarrow \mu ^+\mu ^-)$ will be problematic for the LHT model.

**Fig. 9:**
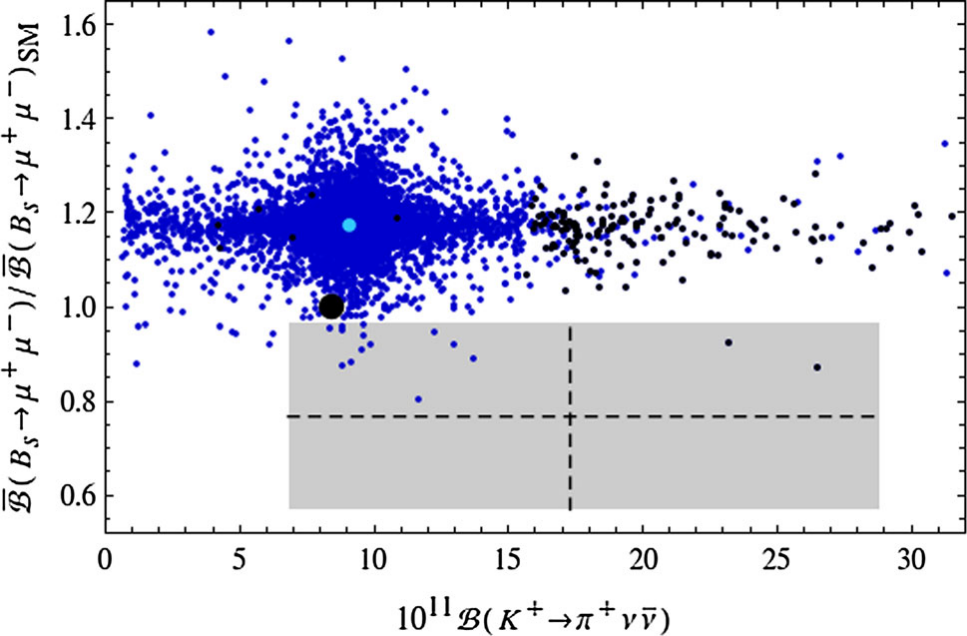
Correlation between $\mathcal {B}(K^+\rightarrow \pi ^+\nu \bar{\nu })$ and $\bar{\mathcal {B}}(B_s\rightarrow \mu ^+\mu ^-)$ in the LHT model for $f=1\, \mathrm{TeV}$. The *large black dot* shows the central SM value for our choice of input parameters, and the *light blue point* shows the contribution from the T-even sector. The experimental $1\sigma$ ranges are displayed by the *grey rectangle* [50, 70]. The black LHT points are excluded by the constraint from $K_L\rightarrow \mu ^+\mu ^-$

The difficulty to suppress $\mathcal {B}(B_s\rightarrow \mu ^+\mu ^-)$ below its SM value is also seen in Fig. 9. Additionally we observe that for the lowest values of $\mathcal {B}(B_s\rightarrow \mu ^+\mu ^-)$ favoured by the data, large enhancements of $\mathcal {B}(K^+\rightarrow \pi ^+\nu \bar{\nu })$ are not allowed.

Even more problematic for the LHT model appear at present the data on $B_d\rightarrow K(K^*) \ell ^+\ell ^-$ as we discussed already in Sect. 3.9.

**Fig. 10:**
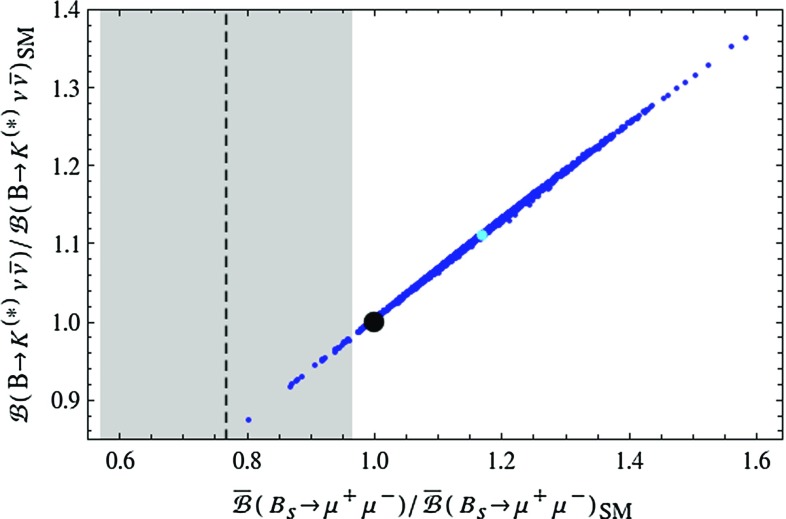
Correlation between $\bar{\mathcal {B}}(B_s\rightarrow \mu ^+\mu ^-)$ and $\mathcal {B}(B\rightarrow K^{(*)}\nu \bar{\nu })$ in the LHT model for $f=1\, \mathrm{TeV}$. The *large black dot* shows the central SM value for our choice of input parameters, and the *light blue point* shows the contribution from the T-even sector. The experimental $1\sigma$ range for $\bar{\mathcal {B}}(B_s\rightarrow \mu ^+\mu ^-)$ is displayed by the *grey band* [50]

### ${B\rightarrow K^{(*)}\nu \bar{\nu }}$

In Fig. 10 we show the correlation between $\bar{\mathcal {B}}(B_s\rightarrow \mu ^+\mu ^-)$ and $\mathcal {B}(B\rightarrow K^{(*)}\nu \bar{\nu })$ in the LHT model. We observe a very strong linear correlation characteristic for models with left-handed flavour changing currents in which the *Z* penguin dominates. We also note as in Fig. 9 that the T-even sector by itself would be in conflict with experiment but the presence of mirror quarks allows still to save the LHT model. Yet, as already seen in Fig. 9, it is difficult to obtain results within $1\sigma$ from the experimental central value.

### Results for scenario B

Let us finally study the pessimistic scenario that no new particles will be discovered at the LHC and all electroweak and Higgs physics observables turn out to be SM-like. In this case the symmetry breaking scale *f* and the mirror fermion masses will be pushed into the multi-TeV range, as discussed in Sect. 4.3.

**Fig. 11:**
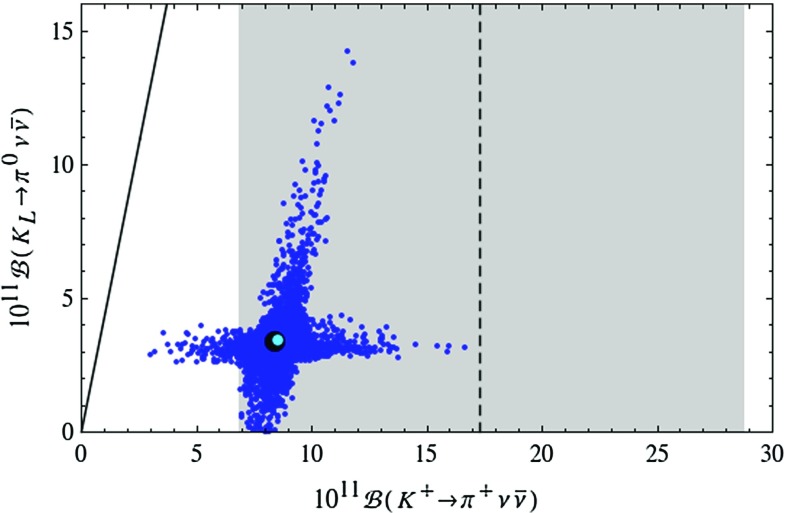
Correlation between the branching ratios of $K^+\rightarrow \pi ^+\nu \bar{\nu }$ and $K_L\rightarrow \pi ^0\nu \bar{\nu }$ in the LHT model for $f=3\, \mathrm{TeV}$. The *large black dot* shows the central SM value for our choice of input parameters, and the *light blue point* shows the contribution from the T-even sector. The experimental $1\sigma$ range for $\mathcal {B}(K^+\rightarrow \pi ^+\nu \bar{\nu })$ is displayed by the *grey band* [70], while the *solid black line* indicates the Grossman–Nir bound [107]

It turns out that in this case rare *K* decays, in particular the $K \rightarrow \pi \nu \bar{\nu }$ decays, are the best channels to observe a sign of the LHT model. As we can see in Fig. 11, significant enhancements of the branching ratios of $K^+\rightarrow \pi ^+\nu \bar{\nu }$ and $K_L\rightarrow \pi ^0 \nu \bar{\nu }$ will still be possible. Again we observe the known two-branch structure. On the horizontal branch $K_L\rightarrow \pi ^0 \nu \bar{\nu }$ remains SM-like, while $K^+\rightarrow \pi ^+\nu \bar{\nu }$ can be enhanced by up to a factor of two. On the second branch the impact on $K^+\rightarrow \pi ^+\nu \bar{\nu }$ is more modest, but $\mathcal {B}(K_L\rightarrow \pi ^0 \nu \bar{\nu })$ can be larger than its SM prediction by up to a factor of four. But again if the present low values of $\mathrm{{Re}}(\varepsilon '/\varepsilon )_\text {SM}$ will be confirmed by more precise lattice calculations, only a suppression of $\mathcal {B}(K_{L}\rightarrow \pi ^0\nu \bar{\nu })$ in Fig. 11 will be allowed and $\mathcal {B}(K^+\rightarrow \pi ^+\nu \bar{\nu })$ will be SM-like.

**Fig. 12:**
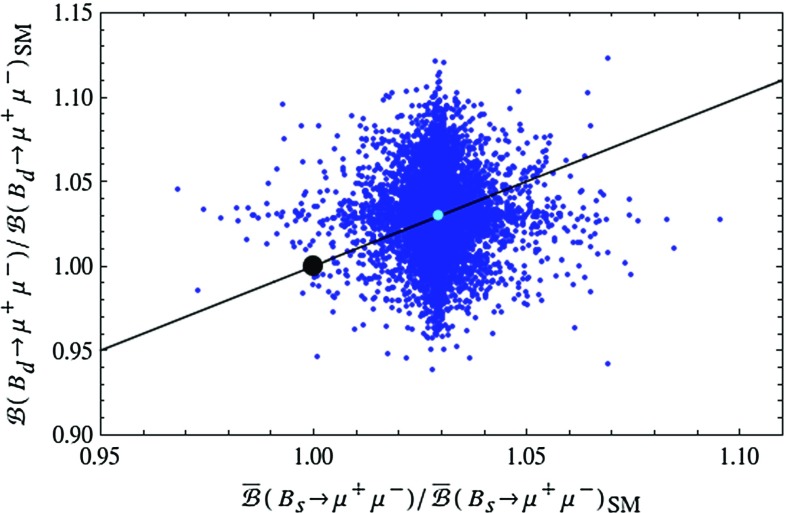
Correlation between $\bar{\mathcal {B}}(B_s\rightarrow \mu ^+\mu ^-)$ and $\mathcal {B}(B_d\rightarrow \mu ^+\mu ^-)$ in the LHT model for $f=3\, \mathrm{TeV}$. The *large black dot* shows the central SM value for our choice of input parameters, and the *light blue point* shows the contribution from the T-even sector

The effects in rare *B* decays on the other hand turn out to be much smaller and, in view of experimental and parametric uncertainties, will be difficult to disentangle from the SM. In Fig. 12 we show the correlation between $\bar{\mathcal {B}}(B_s\rightarrow \mu ^+\mu ^-)$ and $\mathcal {B}(B_d\rightarrow \mu ^+\mu ^-)$ as an example.

It is interesting to see how the LHT effects in rare meson decays scale with the symmetry breaking scale *f*. Naively, the new contributions are suppressed by $v^2/f^2$ with respect to the SM. This is indeed what we see in the T-even sector, displayed by the light blue point in the figures. The case of the T-odd sector is, however, a bit more involved. Firstly, as we increase the mirror quark masses simultaneously with the scale *f*, the size of the loop functions remains unchanged and the only suppression stems from the $v^2/f^2$ prefactor. However, simultaneously the constraints on the T-odd sector from $\Delta F = 2$ observables become weaker, they scale like

94 $$\begin{aligned} \xi ^2 \frac{v^2}{f^2} < \epsilon \end{aligned}$$

with $\xi$ denoting the relevant combination of $V_{Hd}$ elements and $\epsilon \ll 1$ depending on the meson sector in question. The T-odd contributions to $\Delta F = 1$ processes on the other hand scale as

95 $$\begin{aligned} \xi \frac{v^2}{f^2} < \frac{v}{f} \sqrt{\epsilon }. \end{aligned}$$

We conclude that the mirror quark contributions are only linearly suppressed by the scale *f*.

## Summary

In this paper we have presented a new analysis of quark flavour observables within the LHT model. Our analysis takes into account the most recent data from the LHCb experiment, the improvements on CKM parameters and hadronic parameters from lattice QCD and the new lower bounds on the masses of new gauge bosons and mirror quarks. Our main findings are as follows:

- The LHT model agrees well with the data on $\Delta F=2$ observables and is capable of removing some slight tensions between the SM predictions and the data.
- The most interesting departures from SM predictions can be found for $K^+\rightarrow \pi ^+\nu \bar{\nu }$ and $K_{L}\rightarrow \pi ^0\nu \bar{\nu }$ decays, when only constraints from $\Delta F=2$ observables are taken into account. An enhancement of the branching ratio for $K^+\rightarrow \pi ^+\nu \bar{\nu }$ by a factor of two relative to the SM prediction [69] is still possible. An even larger enhancement in the case of $K_{L}\rightarrow \pi ^0\nu \bar{\nu }$ is allowed. But as we have shown in Fig. 6, the recent analysis of $\varepsilon '/\varepsilon$ in the SM [39], based on new results for the non-perturbative parameters $B_6^{(1/2)}$ and $B_8^{(3/2)}$ from lattice QCD [36, 37] and the large *N* approach [38], appears to exclude this possibility at present. Rather a suppression of $K_{L}\rightarrow \pi ^0\nu \bar{\nu }$ is required to fit the data on $\varepsilon '/\varepsilon$. On the other hand as seen in Fig. 7, no significant shifts of $K^+\rightarrow \pi ^+\nu \bar{\nu }$ with respect to SM are allowed.
- NP effects in rare $B_{s,d}$ decays are significantly smaller than in rare *K* decays. Still they can amount to up to a factor of 2 in the $b\rightarrow d$ system and to about $50\,\%$ of the SM branching ratios in $b\rightarrow s$ transitions, like $\mathcal {B}(B_s\rightarrow \mu ^+\mu ^-)$ and $B\rightarrow K^{(*)}\nu \bar{\nu }$.
- More interestingly the pattern of departures from SM expectations for $B_{s,d}$ decays predicted by the LHT model disagrees with the present data. $\mathcal {B}(B_s\rightarrow \mu ^+\mu ^-)$ is favoured by this model to be enhanced rather than suppressed as indicated by the data, and the simultaneous enhancement of $\mathcal {B}(B_d\rightarrow \mu ^+\mu ^-)$ cannot be explained. Furthermore, the LHT model fails to reproduce the $B_d\rightarrow K^{(*)}\ell ^+\ell ^-$ and $R(D^{(*)})$ anomalies observed by the LHCb, BaBar and Belle experiments.

The future of the LHT model depends crucially on the improved experimental values of $\mathcal {B}(B_{s,d}\rightarrow \mu ^+\mu ^-)$ and on the future of the $B_d\rightarrow K^{(*)} \ell ^+\ell ^-$ anomalies. If these anomalies will be confirmed by future more accurate data and theory predictions, then the LHT model is not the NP realised by nature. For this model to survive the flavour tests in the quark sector, the anomalies in question have to disappear. Then also significant enhancements of the branching ratios for $K^+\rightarrow \pi ^+\nu \bar{\nu }$ and $K_{L}\rightarrow \pi ^0\nu \bar{\nu }$ will be possible. Note, however, that these could be forbidden by $\varepsilon '/\varepsilon$ if the future more precise lattice calculations of $B_6^{(1/2)}$ confirm the bound (61).

We have also analysed the case of a higher scale $f= 3\, \mathrm{TeV}$. As seen in Figs. 11 and 12, NP effects are significantly smaller than for $f= 1\, \mathrm{TeV}$. Yet the rare $K\rightarrow \pi \nu \bar{\nu }$ decays still show sizeable LHT effects, which are particularly welcome as in such a scenario an LHT discovery based on direct searches and electroweak and Higgs physics will be difficult. In addition thanks to the pattern of deviations a distinction between the SM, the LHT model, and other NP scenarios on the basis of flavour observables discussed by us should in principle be possible.

In view of these definite findings we are looking forward to improved experimental data and improved lattice calculations. The plots presented by us should facilitate monitoring the future confrontations of the LHT model with the data and help to determine whether this simple model can satisfactorily describe the observables considered by us.

## Footnotes

1 We thank an unknown referee for raising this question.

2 In our conventions $S_{\psi \phi } = -\sin \phi _s$, with the measured value for $\phi _s$ quoted by LHCb and HFAG.

3 Note that the mirror fermions in a doublet are degenerate in mass, up to a small splitting from electroweak symmetry breaking.
